# Red-to-NIR-Fluorescent Graphene Quantum Dots for Biomedical Applications

**DOI:** 10.3390/bios16070386

**Published:** 2026-07-16

**Authors:** Shuyi He, Weichao Liu, Kang Qin, Steven Xu Wu

**Affiliations:** Department of Chemistry, University of South Dakota, Vermillion, SD 57069, USA; shuyihe9703@gmail.com (S.H.); weichao.liu@coyotes.usd.edu (W.L.); kang.qin@coyotes.usd.edu (K.Q.)

**Keywords:** graphene quantum dots, red-to-near-infrared fluorescence, biomedical applications

## Abstract

Graphene quantum dots (GQDs) have attracted extensive interest in biomedical applications because of their favorable physicochemical properties, including environmental friendliness, excellent water solubility, high chemical stability, and facile surface modification. However, most GQDs exhibit fluorescence in the ultraviolet or visible region, which limits their biomedical applications because autofluorescence from biological systems reduces the signal-to-noise ratio in biosensing and bioimaging. Over the past decade, the emission of GQDs has been extended from the UV–visible region into the red-to-near-infrared (NIR) region. Red-to-NIR fluorescence enables higher-resolution imaging and deeper tissue penetration by reducing light scattering and minimizing tissue absorption and autofluorescence. In this review, we summarize recent advances in red-to-NIR-fluorescent GQDs for biomedical applications, including their synthesis, optical properties, surface engineering, and applications in biosensing, bioimaging and theranostics. Finally, we discuss the current challenges and future potential development of the red-to-NIR-fluorescent GQDs.

## 1. Introduction

Thanks to their excellent photoluminescence properties, biocompatibility, water solubility, tunable size, and facile surface modification, graphene quantum dots (GQDs) have attracted widespread research interest across multiple disciplines [1,2,3]. Compared with other carbon-based nanomaterials, such as carbon nanotubes, graphene, and fullerenes, GQDs possess unique, bright, and tunable photoluminescence, which greatly expands the biomedical applications of carbon-based nanomaterials [4]. Typically, GQDs are zero-dimensional carbonaceous nanomaterials consisting of graphene lattices with fewer than ten graphene layers. GQDs are generally defined as graphene fragments with lateral dimensions below approximately 10 nm or less. However, depending on the synthesis method and the criteria adopted in different studies, some reported GQDs exhibit lateral sizes of up to approximately 20 nm [5,6]. The unique structure endows GQDs with tunable photoluminescence, excellent photostability, and remarkable multiphoton excitation capabilities [7,8].

Although their excellent photoluminescence properties make GQDs attractive for applications in biomedical fields, the origin of their fluorescence remains incompletely understood, which limits the rational design of GQDs with desired emission wavelengths. Most GQDs exhibit absorption and fluorescence emission in the ultraviolet, blue, or green spectral regions [9,10]. Fluorescence in these spectral regions suffers from strong autofluorescence from biological samples, which significantly reduces the signal-to-noise ratio and consequently limits detection sensitivity. To minimize biological autofluorescence, red-to-near-infrared (NIR) fluorescence is preferred because it provides lower background interference and greater tissue penetration [11]. However, the development of red-to-NIR GQDs is still at an early stage because the mechanisms responsible for red-to-NIR emission are complex and have not yet been fully elucidated. Numerous factors have been proposed to influence red-to-NIR fluorescence, including particle size, sp^2^ domain size, surface hydrophilicity, and heteroatom doping. Therefore, summarizing the recent progress in red-to-NIR GQDs is essential for understanding their optical properties and promoting their biomedical applications. In this review, we provide a comprehensive overview of the recent advances in red-to-NIR-fluorescent GQDs, with an emphasis on their synthesis, optical properties, surface engineering, and biomedical applications.

## 2. Development of GQDs for Biomedical Applications

Graphene is a carbon allotrope derived from graphite, consisting of a single layer of atoms arranged in a hexagonal nanolattice. Since its isolation by mechanical exfoliation, graphene has attracted tremendous attention because of its unique two-dimensional sp^2^-hybridized structure and exceptional electrical, optical, thermal, and mechanical properties [12]. Graphene’s superior properties, such as excellent electrical conductivity, chemical stability, optical transparency, thermal conductivity, and excellent hydrophobicity, have enabled its wide application [13].

One of the major research directions is to convert two-dimensional graphene sheets into zero-dimensional GQDs. Although GQDs are often grouped within the broader category of carbon dots (C-dots), they possess distinct structural characteristics [14]. GQDs consist predominantly of crystalline sp^2^-hybridized graphene domains, whereas conventional C-dots usually contain mixed sp^2^/sp^3^ carbon structures with a more amorphous framework. In addition, conventional C-dots are typically smaller than 10 nm [15], whereas GQDs and their derivatives generally exhibit lateral dimensions ranging from approximately 3 to 20 nm [16]. Owing to their low toxicity, stable photoluminescence, excellent water solubility, chemical inertness, and facile surface modification, GQDs have attracted increasing attention in biomedical applications.

The biomedical applications of GQDs have expanded rapidly over the past decade. Early studies primarily focused on biosensing by exploiting the fluorescence-quenching capability of GQDs and their versatile surface functional groups. In 2011, Zhao et al. reported the sensitive detection of single-stranded DNA and proteins using DNA probe-functionalized GQDs [17]. Subsequently, GQD-based biosensors were developed for the detection of ATP [18,19], Hg^2+^ [20], Cu^2+^ [21], trypsin [22], glucose [23], protein kinase [24], and other biologically important analytes. Beyond biosensing, GQDs have also been explored for DNA cleavage [25], promotion of i-motif structure formation [26], drug delivery [27,28], enzyme-mimicking catalysis [29], and photodynamic therapy [30], indicating their versatility as multifunctional biomedical nanomaterials.

Considerable efforts have also been devoted to bioimaging applications. Over the past decade, GQDs have been widely investigated as fluorescent probes for both in vitro and in vivo imaging owing to their excellent photostability and favorable biocompatibility [27,31]. Their biosafety has also been extensively evaluated through in vitro [32] and in vivo studies [33], demonstrating generally low toxicity. To improve imaging performance, researchers have reported GQDs exhibiting upconversion photoluminescence [34] and two-photon fluorescence imaging capabilities with penetration depths of up to 1800 µm [35]. More recently, increasing attention has shifted toward red-to-near-infrared (NIR) fluorescent GQDs because NIR fluorescence experiences lower interference from tissue autofluorescence, reduced light scattering, and greater tissue penetration [36].

## 3. Preparation of GQDs

### 3.1. Bottom-Up Method

The bottom-up strategy offers significant advantages for controlling the morphology and structure of GQDs through chemical reactions or precursor assembly, enabling the design of GQDs with desired properties. This approach is simpler, more cost-effective, and less time-consuming than top-down methods. Additionally, it requires fewer resources, utilizes readily available raw materials, and is well suited for large-scale preparation. Below, we summarize representative bottom-up methods for preparing red-to-NIR-fluorescent GQDs in Table 1.

#### 3.1.1. Hydrothermal/Solvothermal Treatment

Among the numerous methods for GQD synthesis, hydrothermal/solvothermal treatment is the most widely used. Hydrothermal synthesis has a significant influence on the size of GQDs. D. Pan et al. first applied hydrothermal technology to synthesize GQDs. The reaction mechanism is that the epoxy groups and carbonyl groups in the graphene sheet structure are easily destroyed during the hydrothermal reduction process [48]. Huang et al. successfully prepared GQDs with the maximum emission peak at 700 nm, which could be tuned by changing the alkyl chain length of precursors. It demonstrated that decreasing the bandgap energy, in other words, increasing the degree of conjugation, results in a red shift of the absorption and emission peaks [37]. Zulhanip et al. reported a one-step hydrothermal cutting method for synthesizing GQDs. The formation of the synthesized GQDs could be controlled using only the alkaline additive sodium hydroxide (NaOH), which acts as both an alkaline-induced fragmentation agent and a surface modifier, achieving surface functionalization [49]. Ferla et al. synthesized CQDs via a hydrothermal method using different precursors (e.g., p-phenylenediamine, citric acid, and urea) and solvents, and found that the process produced red-emitting CQDs with ~600 nm fluorescence but with low yield and complex mixtures, indicating limitations in synthetic efficiency and purification [50]. Kansara et al. synthesized N-, S-, and B-doped graphene quantum dots using a hydrothermal method and systematically compared their structures, optical properties, and biocompatibility. They found that N-doped GQDs had the highest fluorescence quantum yield and the greatest potential for biomedical applications [51]. Yu et al. prepared GO- and synthesized N-doped GQDs via a hydrothermal method. They found that increasing the GO synthesis temperature enhanced oxygen adsorption, thereby increasing the GQD yield and improving the photoluminescence intensity by approximately 20% [52].

#### 3.1.2. Microwave-Assisted Technique

Microwave radiation consists of electromagnetic waves with wavelengths between infrared and radio waves, which can provide sufficient energy for breaking chemical bonds. Microwave-assisted synthesis is considered to be a fast and economical method, which can effectively reduce the reaction time while providing uniform heating and helping to produce GQDs of uniform size [53]. Unlike the hydrothermal method, which usually takes several hours, the production time of GQDs through microwave-assisted synthesis can be shortened. Hasan et al. reported a single-step microwave-assisted hydrothermal method to prepare NS-GQDs from glucosamine, which contained amine linkages and abundant oxygen-containing functional groups. The reported NS-GQDs showed a high quantum yield of up to 60% [38]. Le et al. synthesized fluorescent GQDs from jackfruit seed starch via a one-step hydrothermal method and demonstrated their antioxidant, anti-inflammatory, photocatalytic dye degradation, and enhanced antibacterial activities when combined with CuO nanoparticles [54]. Kadyan et al. synthesized fluorescent GQDs via a microwave-assisted green method using Azadirachta indica (neem) leaf extract, obtaining spherical GQDs (~5.6 nm) with stable photoluminescence [55]. Roch et al. synthesized carbon dots by treating trinitropyrene (TNP) in DMF using a microwave-assisted solvothermal method, but found that the main fluorescence of the samples originated from the generated molecular fluorophores rather than the actual carbon dots, with a quantum yield of about 20% [56]. Mohamed et al. prepared GQDs using sugarcane bagasse and sugarcane bagasse pith via carbonization and microwave pyrolysis and found that they exhibited good antioxidant and anti-inflammatory activities with low toxicity, making them suitable for biomedical applications [57].

### 3.2. Top-Down Method

The earliest GQDs were developed using the top-down method. The top-down approach is very effective for discovering new materials and studying their structure and properties. Generally, the main principle of the top-down method is to cleave or break down bulk carbonaceous materials such as graphite, graphene, graphene oxide, and carbon fibers via hydrothermal cutting [58], solvothermal cutting [31], microwave-assisted cleavage [59], chemical exfoliation [60] and electrochemical scissoring [61]. Here, we summarize some representative top-down methods used to prepare red-to-NIR-fluorescent GQDs in Table 2.

#### 3.2.1. Electrochemical Exfoliation

Electrochemical exfoliation is a versatile method for preparing GQDs, involving the use of an electrochemical cell to exfoliate bulk graphite into graphene layers, which are then further broken down into quantum dots. This approach is efficient and environmentally friendly and allows for precise control over the size and functionalization of GQDs [69]. Tan et al. proved that the size of the sp^2^ domains of GQDs had a significant impact on the local energy gap and the emission wavelength of fluorescence. The paper also reported an electrochemical synthesis process by exfoliating graphite in K_2_S_2_O_8_ solution to prepare uniform small-sized red-fluorescent GQDs [62]. In Zhang’s study, GQDs were synthesized via electrochemical exfoliation of graphite under constant-current conditions, enabling scalable production with controlled size and surface functionalization [70].

#### 3.2.2. Acidic Exfoliation

Acidic exfoliation and oxidation of large-sized graphene into small GQDs represent one of the earliest methods for GQDs synthesis. Activated carbon, graphene oxide sheets, VCX-72 carbon black, and other carbon materials have been widely used for the mass production of GQDs. At the same time, by-products (such as inorganic salts and acids) inevitably attach oxygen-containing functional groups to the prepared GQDs, which improves their hydrophilicity but also introduces structural defects. Nitric acid oxidation is another simple and convenient approach for preparing GQDs [71]. Shao et al. reported a new method to fabricate bright red-emitting GQDs by increasing the concentration of nitric acid (14.6 M) to sufficiently oxidize the surface and efficiently dope nitrogen (4.31%) [63]. Zarghami et al. synthesized Co- and Nd-doped GQDs via citric acid thermal decomposition and found that doping introduced trap levels within the bandgap, thereby significantly enhancing upconversion luminescence efficiency (41% for Co doping and 100% for Nd doping). These GQDs showed promising potential for bioimaging and drug delivery [72].

#### 3.2.3. Radical-Assisted Exfoliation

Radical-assisted exfoliation is an efficient, simple, environmentally friendly, and scalable method for synthesizing GQDs. By taking advantage of abundant hydroxyl radicals generated through the Fenton reaction under the conditions of UV light [25] and ultrasonic irradiation [73], graphene-based materials can be oxidized to GQDs. Nevertheless, the limitation of this method is that the initial addition of Fe^2+^ may quench the fluorescence of GQDs. Ke et al. improved this method by replacing Fe^2+^ in Fenton’s reagent with NH_4_OH and thiourea, producing GQDs/GO with moderate quantum yields (1–10%), nanosecond fluorescence lifetimes, and excitation-independent emissions in four different colors, including red-emitting GQDs/GO. They also demonstrated that GQDs exhibited tunable fluorescence wavelengths depending on particle size [64].

Taken together, the preparation of red-to-NIR-fluorescent GQDs should be chosen according to the required optical properties and biomedical applications. Bottom-up methods, including hydrothermal/solvothermal and microwave-assisted methods, are more flexible with respect to precursor selection, heteroatom doping, and surface modification, which facilitates the tuning of emission wavelength and quantum yield. However, product yield and reproducibility can be strongly affected by the reaction conditions, precursor composition, and purification process. In comparison, top-down methods are directly based on graphene-related carbon materials and can preserve graphitic domains, but they often involve strong oxidants, acids, electrochemical exfoliation, or additional cutting processes, which may increase the cost, environmental impact, and batch-to-batch variation. Therefore, for biomedical applications, the synthetic method should be chosen by considering optical performance, yield, reproducibility, cost, scalability, and environmental friendliness.

## 4. Characterization

### 4.1. Size and Shape

GQDs are zero-dimensional quantum dots with a small nanometer-scale (2–5 nm) lateral dimension and only 1–3 graphene layers. They show excellent water solubility due to the presence of abundant oxygen-containing functional groups on the edge and basal plane. GQDs are mostly disc-shaped with sp^2^-hybridized carbon, whereas carbon dots are mainly spherical and contain predominantly sp^3^-hybridized carbon [74]. The sizes of GQDs are usually controlled by the synthesis method. GQDs have different shapes including elliptical, triangular, quadrate, and hexagonal structures [58].

**Figure 1:**
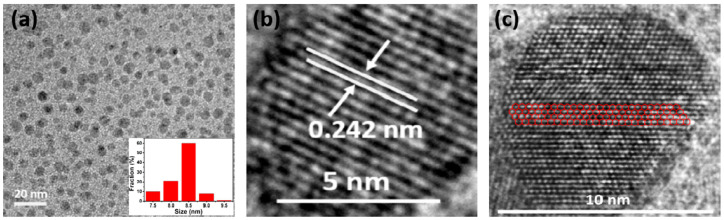
(**a**) TEM image of GQDs and size distribution of GQDs. (**b**) HRTEM images with measured lattice spacing and sp^2^ domain (**c**) of GQDs. Reproduced with permission from Li et al. [75], *ACS Applied Materials & Interfaces*; published by American Chemical Society, 2017.

To obtain information on the size and morphology of GQDs, multiple techniques can be used for their characterization, including transmission electron microscopy (TEM), scanning electron microscopy (SEM), and atomic force microscopy (AFM). TEM can be used to observe the morphology of GQDs and their atomic-scale structures. For example, as shown in Figure 1, TEM images showed that GQDs were well dispersed, with a size distribution of about 7.5–9.5 nm and an average diameter of 8.5 nm [75]. High-resolution TEM (HRTEM) images showed that most GQDs exhibited a uniform atomic arrangement and high crystallinity. The lattice spacing of GQDs was 0.242 nm, which was consistent with the lattice fringe characteristics of graphene [76]. GQDs were reported to contain relatively large and continuous sp^2^ domains, indicated by the well-ordered hexagonal carbon lattice [77]. The blurred boundaries were attributed to the introduction of oxygen-containing groups. HRTEM therefore provides valuable information on the structural integrity, lattice structure, crystallinity, and structural defects of GQDs.

**Figure 2:**
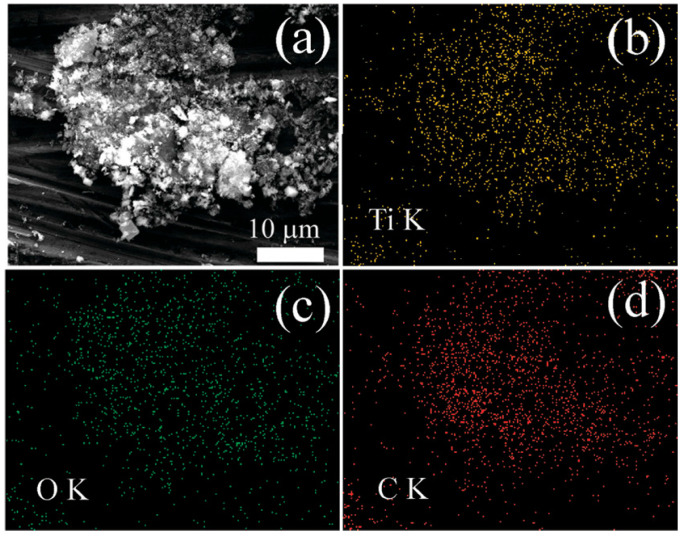
(**a**) SEM image of rutile TiO_2_/GQDs composites and mapping of its surface elements: (**b**) Ti, (**c**) O, and (**d**) C. Reproduced with permission from Zhuo et al. [78], *ACS Nano*; published by American Chemical Society, 2012.

SEM is usually used to analyze the surface morphology of the samples, and the sample composition can be analyzed using an energy-dispersive X-ray spectroscopy (EDS) detector attached to an SEM. It can be used to observe and analyze the surface morphology of GQDs and analyze the content of elements in the sample by EDS. As shown in Figure 2, the prepared TiO_2_/GQD nanocomposite exhibited a rutile structure, and the Ti, O, and C elements were evenly distributed throughout the material [78].

**Figure 3:**
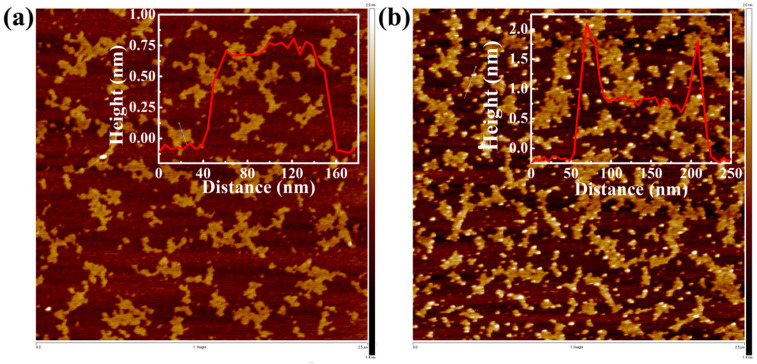
AFM images of (**a**) TMPyP and (**b**) NGQDs•TMPyP. Reproduced with permission from Peng et al. [79], *ACS Sensors*; published by American Chemical Society, 2018.

The surface morphology and thickness of GQDs could be precisely measured by AFM. In Peng’s work, shown in Figure 3, the AFM images clearly revealed that NGQDs successfully combined with MnII (TMPyP), as evidenced by the significant changes in the surface topography and height of the GQDs [79].

### 4.2. Function Groups

GQDs possess a high degree of crystallinity, and their interlayer spacing can be modified by introducing different functional groups at the edges of the GQDs during synthesis [80]. To better understand the functional groups on the GQD surface, Fourier-transform infrared spectroscopy (FTIR), Raman spectra, X-ray diffraction (XRD), and X-ray photoelectron spectroscopy (XPS) are often applied.

**Figure 4:**
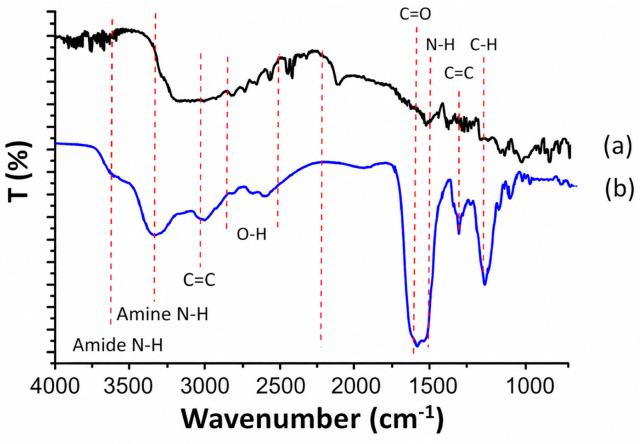
FTIR spectra of the precursors of GQDs (L−glutamic acid) (a) and prepared GQDs (b). Reproduced in part with permission from Wu et al. [42], *Journal of Materials Chemistry C*; published by Royal Society of Chemistry, 2013.

FTIR is used to analyze the molecular structure and functional groups of GQDs, and to identify the types of functional groups present, although it cannot quantitatively determine their concentration. FTIR is often used to identify the types of functional groups contained in the prepared GQDs and compared with the precursors. For instance, from FTIR, it was not only proven that GQDs were formed by the presence of C=C stretching of graphite at the 3000 cm^−1^ and 1500 cm^−1^ peaks [81], but it also indicated that the GQDs obtained carboxyl groups, amine groups, and newly formed amides (Figure 4) [42].

**Figure 5:**
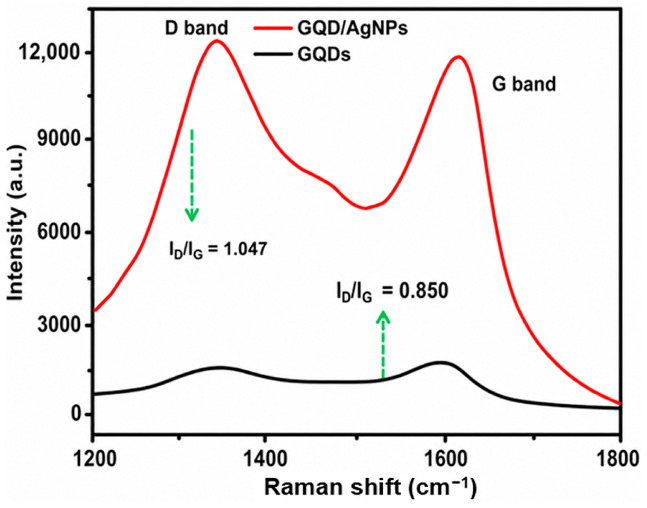
Raman spectra of GQD/AgNPs and GQDs. The green arrows indicate the D-to-G band intensity ratios for GQD/AgNPs and GQDs. Reproduced in part with permission from Chen et al. [83], *ACS Biomaterials Science & Engineering*; published by American Chemical Society, 2017.

Raman spectra are based on the analysis of the Raman scattering effect that occurs when light passes through the sample. By analyzing the frequency, intensity, peak position, and half-width of the Raman spectrum, the number of layers, defects, crystal structure, and phonon energy band of GQDs could be obtained. It is an important technique for GQD testing and analysis [82]. GQDs with defects will have Raman D peaks near 1350 cm^−1^. The intensity ratio of D peak to G peak (I_D_/I_G_) and half width of G peak are generally used to characterize the density of defects in GQDs. For example, Chen et al. used Raman spectroscopy to characterize GQDs and GQD/AgNP hybrids, which both showed D and G bands at about 1349 and 1606 cm^−1^ (Figure 5), respectively. Due to the surface-enhanced Raman scattering effect of AgNP, the intensity of D and G bands of GQD/AgNP was about 10 times higher than that of GQDs. In addition, the D/G intensity of GQD/AgNP hybrids was higher than that of GQDs, indicating that GQD/AgNP hybrids had fewer oxygen-containing functional groups and a lower degree of sp^2^ to sp^3^ carbon conversion than GQDs. The increase in the intensity of the D-band of the GQD/AgNP hybrid illustrates the existence of sp^3^ defects located in the sp^2^ carbon network [83].

**Figure 6:**
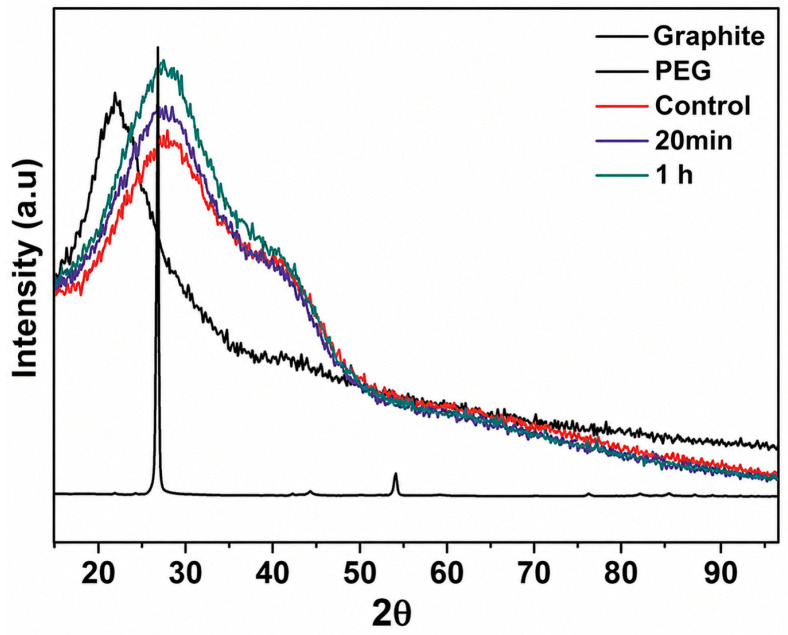
XRD patterns of pure graphite, PEG, control GQDs, 20 min-treated and 1 h- treated GQDs. The two black curves correspond to graphite and PEG, respectively; graphite can be identified by its sharp diffraction peak at 26.95°. Reproduced in part with permission from Narasimhan et al. [68], *RSC Advances*; published by Royal Society of Chemistry, 2017.

XRD is an important method of materials research, mainly used to characterize crystal structure, crystal plane spacing, lattice parameters, and crystallinity. It can analyze and evaluate the reduction degree, layer spacing, and defects of GQDs. As shown in Figure 6, the XRD spectrum of pure graphite has sharp peaks at 002 (2θ = 26.95°) and 004 (2θ = 54.9°) [68,84]. GQDs had the same broad diffraction peak at about 26.95°, which suggested that the lattice spacing was similar to that of pure graphite and confirmed the existence of polycrystalline GQDs [85]. In GQDs, the broad peak in the spectrum corresponds to the turbostratic band of the disordered graphene layer, which is associated with the increase in oxidation and the presence of polyethylene glycol (PEG) [86].

X-ray photoelectron spectroscopy (XPS) is a widely used surface analysis technique that employs X-ray irradiation to eject photoelectrons from a material. By measuring the kinetic energy of these emitted electrons, the binding energy of electrons in different atomic orbitals can be determined. XPS provides important information about the elemental composition, chemical states, and surface functional groups of materials, typically within a depth of several nanometers. Therefore, it is commonly used to confirm the presence of elements, analyze heteroatom doping, and identify chemical bonding configurations on the surface of GQDs [87,88].

### 4.3. Photoluminescence (PL)

#### 4.3.1. Mechanism

Photoluminescence has always been one of the most attractive properties of GQDs. Unlike graphene sheets, which do not exhibit photoluminescence because of their infinitely large sp^2^ domains that result in a zero bandgap, GQDs exhibit strong photoluminescence. The photoluminescence of GQDs is affected by many factors, such as size, pH, solvent, and the synthesis method. The photoluminescence mechanism is generally classified into two categories: intrinsic-state emission and defect-state emission. These two mechanisms not only affect each other but may also change the energy gap of the π–π electrons in the sp^2^ domains, thereby controlling the PL of GQDs. Ensemble photophysical measurements have shown that the optical properties of GQDs depend on the synthesis method. Meanwhile, carbon excitons, emission traps, quantum confinement effects, aromatic structures, oxygen-containing groups, zigzag edge states, and edge defects can also influence their photoluminescence [89,90,91,92]. Different from the traditional view that PL emission depends on the inherent size of GQDs, studies have shown that GQDs can exhibit excitation-independent upconversion and downconversion photoluminescence. At the same time, the photocatalytic activity of GQDs is related to their crystal structure [78]. We should not only consider the electronic transition in simplified monolayer GQDs and their dimers, but also the effects of their interlayers, which may contribute to their edge functionalization and the absorption and emission spectra of GQDs. I.K. Petrushenko et al. used borazine (B_3_N_3_) doping with stable bilayer QDs in different ways to modify their electronic properties and analyze their optical properties. Their results suggested that interlayer interactions should also be considered when designing graphene-based optoelectronic applications [93]. These quantum dots offer the advantages of low cost and environmental friendliness and have broad application prospects in the fields of biosensors and bioimaging.

#### 4.3.2. Fluorescence Quantum Yield

Quantum yield is defined as the number of emitted photons relative to the number of absorbed photons. Pristine GQDs reported in early studies generally exhibited limited quantum yields (less than 5%) [94]. With the rapid development of nanoscience, more and more methods to improve quantum yield have been proposed. Doping heteroatoms is one of the commonly used methods. By doping heteroatoms, the energy bandwidth and electron local density of GQDs were changed, thereby greatly improving the quantum yield of GQDs [95]. For example, Nair et al. used H_2_SO_4_ as the source of sulfur doping and KMnO_4_ as the oxidant to produce green fluorescent S-GQDs. XPS and FTIR analysis confirmed that sulfur doping can indeed increase the fluorescence intensity, lifetime, and quantum yield (27.8%) of GQDs [96]. In addition, it is reported that doping N [97], B [98], Cl [99], and other atoms in GQDs could improve the quantum yields so that they could be used in different fields. Polyatomic doping to produce a synergistic effect would be another good way to improve quantum yield. Wang et al. prepared S- and P-doped GQDs with a high quantum yield by taking advantage of the electronegativity difference between P and S. The valence electrons of P in the third shell could be more readily removed than those of S, and the higher surface electron density of O atoms on GQDs further promoted their synergistic effect. Therefore, S- and P-GQDs show higher QY than single-element-doped GQDs [100].

Quantum yield also depends on the synthesis pathway and surface modification. By covalently modifying a coumarin derivative onto the surface of GQDs, the fluorescence quantum yield was enhanced (18%) [101]. Narasimhan et al. used a nanosecond pulsed laser to ablate highly oriented pyrolytic graphite (HOPG) to prepare high-quality water-soluble GQDs. They found that changing the reflow time at 200 °C can affect the quantum yield of GQDs. Compared with the control group (QY of 24.9%), GQDs with 20 min reflux have a higher quantum yield (47.16%). However, when the reflux time was further increased to 1 h, the quantum yield decreased to 12.8%, possibly because of changes in pH [68]. In Lee’s work, the photoreduced rGOQDs exhibited excitation-independent blue fluorescence emission (approximately 440 nm) and the quantum yield increased to approximately 19.7%, about 10-fold higher than that of the original GOQDs. Furthermore, these quantum dots exhibit low cytotoxicity and can be used for fluorescence imaging of HEK293A cells, demonstrating their potential applications in bioimaging and optoelectronic devices [102].

#### 4.3.3. Fluorescence Lifetimes

The fluorescence lifetime of GQDs refers to the time that the excited-state electrons remain before returning to the ground state through photon emission [103]. Analysis of fluorescence lifetime provides important information for understanding the optoelectronic and catalytic properties of GQDs [104]. The fluorescence lifetime of GQDs is affected by the synthesis method and surface modification. GQDs synthesized from the bottom-up method have lower heterogeneity than GQDs synthesized from the top-down method, and thus have a more uniform fluorescence lifetime instead of the distribution of lifetimes [105]. The fluorescence lifetime of GQDs gradually decreases due to the introduction of new carboxyl groups on the surface/edge of GQDs [68]. In addition, GQDs with more layers are usually brighter but have shorter fluorescence lifetimes [106].

#### 4.3.4. Factors in the Red Shift of Emission

Near-infrared imaging has particularly unique advantages for in vivo imaging, including greater penetration depth, lower background noise, and less photodamage and phototoxicity [107]. How to prepare red-to-NIR-fluorescent GQDs has always been a major topic of great concern. Here, we summarize some factors that could cause the red shift of GQDs. It has been reported that nitrogen doping not only generated near-infrared emissive GQDs but also enhanced light stability. This can be attributed to the ability of nitrogen dopants to modify the electronic structure of GQDs. Graphitic N can tune the π-conjugated sp^2^ carbon framework, whereas pyridinic and pyrrolic N can introduce edge- or surface-related defect states. These newly formed electronic states may reduce the effective energy gap and create lower-energy emissive pathways, leading to red-shifted fluorescence. In addition, co-doping with heteroatoms such as S can further promote charge redistribution and surface-state formation, which may improve fluorescence efficiency. The study also pointed out that both doped and undoped GQDs exhibit size-dependent photophysical properties [108]. Xu et al. used single-particle spectroscopy to study single GQDs for the first time. The study found that after aging in ambient air, almost all measured single GQDs showed a certain degree of red-shifted photoluminescence, which may be due to the desorption of water, and can be restored with the addition of water. In addition, single GQDs did not show single-photon emission, but their stable fluorescence was not affected by photoluminescence intermittent and bleaching effects, indicating that they have great advantages as a fluorescent probe over traditional organic dyes and semiconductor quantum dots [106]. Using density functional theory (DFT) and time-dependent calculations (TDDFT), Mahasin et al. found that the PL of GQDs can be sensitively adjusted based on size, edge configuration, shape, attached chemical function, heteroatom doping, and defects. The PL of GQDs is essentially determined by the embedded sp^2^ clusters segregated by sp^3^ carbon. At the same time, the edge of the armchair and the doping of pyrrole nitrogen could cause a blue shift of GQD PL emission, while chemical functions and defects will cause a red shift [109].

The oxygen-containing groups in GQDs also influence the emission wavelengths of GQDs. For instance, Hasan et al. modified nitrogen-doped GQDs with ozone; the size of ozone-treated NGQDs (Oz-NGQDs) was reduced to 5.5 nm, and both the lattice structure and I_D_/I_G_ Raman ratio changed significantly. This is due to the introduction of oxygen-containing functional groups. As the atomic/weight percentage and structure of oxygen atoms change, the absorption of Oz-NGQDs in the ultraviolet–visible (UV-vis) region decreases, and the fluorescence in the visible and near-infrared (NIR) regions gradually increases [40].

## 5. Surface Modification

### 5.1. Non-Covalent Modification

The non-covalent modification of GQDs provides an effective approach for reversible functionalization while preserving the original GQD structure. It is often used in applications requiring reversible ligand exchange [110] and maintains the original structure of GQDs to maintain their fluorescence properties [111]. Jeong et al. compared the adsorption affinities of GQDs towards ssDNA, phospholipids, and protein-like polymers with different degrees of oxidation (summarized in Table 3). The study found that the oxygen content of GQDs determined their adsorption affinity towards ssDNA, which could be adsorbed on the surface of no- and low-ox GQDs, but not on the surface of medium- or high-ox GQDs [112].

**Figure 7:**
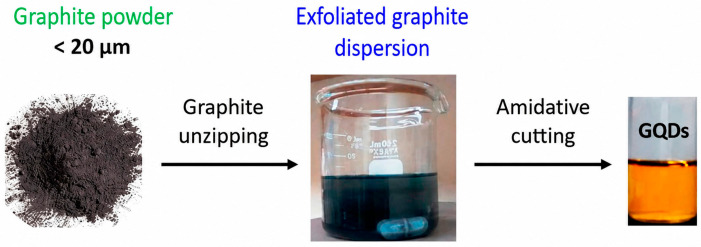
Synthesis process and characterization of GQDs: two-step preparation process of multiple-color GQDs under UV excitation. Reproduced in part with permission from Sapkota et al. [114], *ACS Applied Materials & Interfaces*; published by American Chemical Society, 2017.

One common strategy for preparing red-fluorescent GQDs is to couple organic dyes with GQDs. This approach usually requires surface activation and covalent surface functionalization to enable the attachment of organic dyes. However, it does not always result in a consistent improvement in fluorescence quantum yield. In contrast, non-covalent functionalization involves fewer synthetic steps. Liu et al. first proposed modifying GQDs with chelating ligands dibenzoylmethane (DBM) and 1,10-phenanthroline (Phen) through non-covalent functionalization, followed by coordination with Eu (III). Not only was the problem of poor water solubility of Eu (III) solved, but NIR-fluorescence GQDs with high quantum yield can also be obtained [113]. Sapkota et al. prepared GQDs with tunable optical properties and enhanced their water stability through non-covalent surface functionalization [114] (Figure 7).

### 5.2. Covalent Modification

#### 5.2.1. EDC/NHS Conjugation

The 1-(3-Dimethylaminopropyl)-3-ethylcarbodiimide hydrochloride (EDC)/3-(maleimido)propionic acid N-hydroxysuccinimide ester (NHS) cross-linking method is widely used for the surface modification of nanoparticles. The principle of the reaction is that EDC helps form an amide bond between the amino group and the carboxyl group, which itself does not actually become part of the cross-linking. EDC is first coupled with a carboxyl group to form an O-acylisourea intermediate. This activated intermediate product is attacked by the -NH_2_ group, thereby forming an amide crosslink, and the activated intermediate product can be eliminated and washed away. Using this method, Yu et al. conjugated coumarin derivatives onto GQDs to prepare for detecting CN^-^, hydroquinone (HQ), and ascorbic acid (AA) in live cells and fresh fruits [101].

#### 5.2.2. One-Pot Hydrothermal Reaction

**Figure 8:**
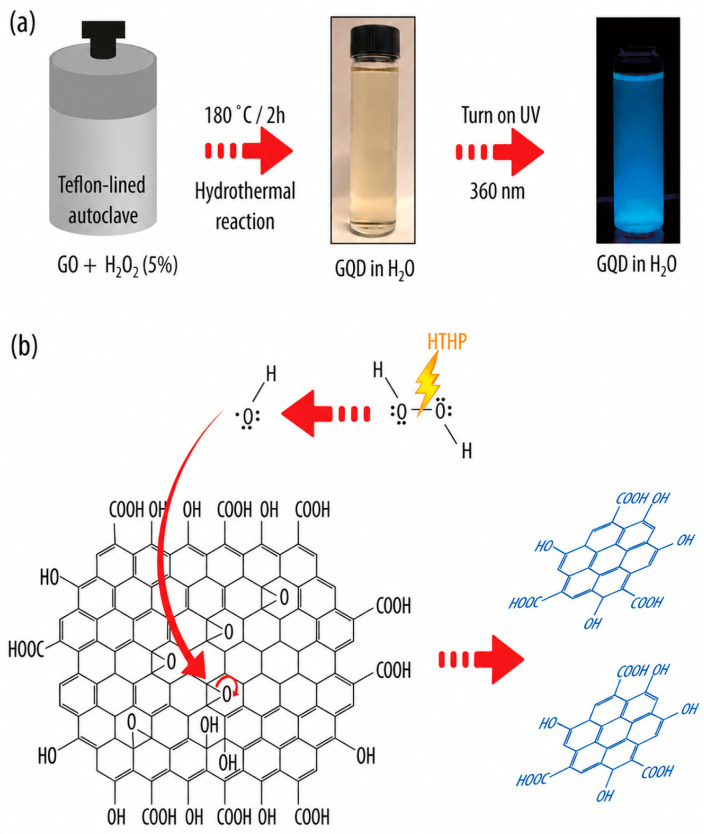
GQD synthesis: (**a**) GQD synthesis from GO and digital photographs of GQDs in water with and without 360 nm wavelength UV excitation. (**b**) Mechanism of hydroxyl radical (•OH) formation and its attack on epoxy groups on GO, as indicated by the curved red arrow, to synthesize GQDs under hydrothermal high-temperature high-pressure (HTHP) condition. Reproduced with permission from Halder et al. [116], *ACS Applied Bio Materials*; published by American Chemical Society, 2018.

Besides conventional covalent modification, a facile one-pot hydrothermal method has also been developed for the surface modification of GQDs to obtain different functionalities. For example, Lin et al. developed a robust modification method to prepare GQDs with different fluorescence properties, tunable hydrophilicity–hydrophobicity, and organelle targeting ability [115]. Halder et al. reported a one-pot, hydrogen peroxide-assisted hydrothermal method for synthesizing fluorescent GQDs, yielding biocompatible nanoprobes without the need for harsh chemicals or additional purification [116] (Figure 8).

#### 5.2.3. Coating Reaction

**Figure 9:**
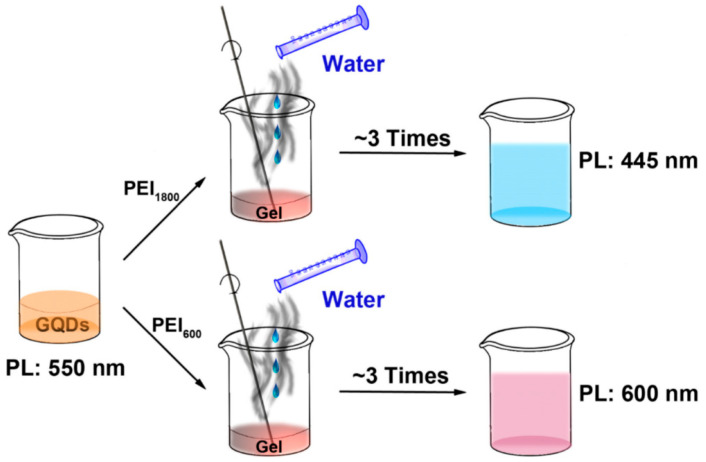
Schematic illustration of the synthesis of different-molecular-weight PEI-coated GQDs. Reproduced with permission from Gao et al. [67], *ACS Applied Materials & Interfaces*; published by American Chemical Society, 2017.

When designing and preparing PL-tunable GQDs, it is necessary to keep the size of the carbon bone structure and the internal graphene unchanged. The coating reaction of GQDs is an ideal method. By coating materials on GQDs, a core–shell structure was formed to protect the internal GQDs, which maintained the intrinsic luminescence characteristics and stabilized GQDs through the passivation effect. Various coating materials have been applied for the surface modification of GQDs. For example, Liu et al. used oligomeric poly(ethylene glycol) diamine (PEG1500N) as a coating material to improve the dispersibility of GQDs in water [117]. Nurunnabi et al. used polydopamine (PDA) as a coating material to improve the biocompatibility and photostability of GQDs [118]. Gao et al. successfully prepared blue-, yellow-, and red-emission GQDs by coating them with polyethylenimine (PEI) of different molecular weights. They mixed GQDs PEI in ultrapure water and heated the mixture to boiling temperature. When a gel formed, ultrapure water was added to prevent drying and burning. This process was repeated three times to obtain PEI-coated GQDs (Figure 9). Through IR spectroscopy and theoretical calculation, it was found that the amidation reaction between the carboxyl group and the amide functional group played an important role in the coating process [67].

## 6. Biomedical Applications

Due to their diverse surface chemical properties, strong photoluminescence, excellent electrical properties, outstanding chemical inertness, and biocompatibility, graphene quantum dots have potential applications in bioimaging, biosensing, antibacterial applications, and drug delivery [119,120]. In the work of Song et al., S- and N-doped graphene quantum dots showed good biocompatibility with human monocytes and macrophages, and can be degraded by enzymes and cells, supporting their safe use in biomedical applications [121].

### 6.1. Sensors for Small Molecules

**Figure 10:**
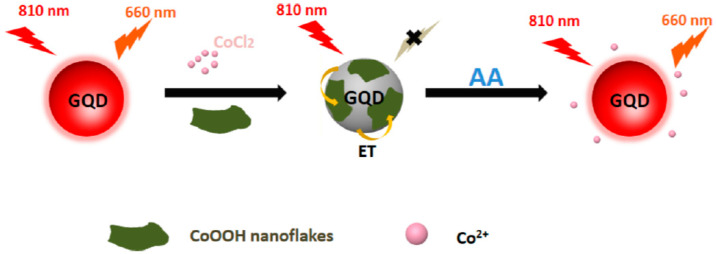
Schematic Illustration of the design and principle of AA detection using NIR GQD/CoOOH nanoprobe. The crossed lightning symbol indicates the quenching of NIR fluorescence by CoOOH nanoflakes, while the restored fluorescence indicates the reduction of CoOOH to Co^2+^ by ascorbic acid. Reproduced with permission from Feng et al. [122], *Analytical Chemistry*; published by American Chemical Society, 2017.

**Ascorbic Acid (AA):** AA is an essential biomolecule found in a wide range of biological fluids and food products. Accurate determination of AA is therefore important for clinical diagnosis, food quality assessment, and pharmaceutical analysis. By taking advantage of two-photon fluorescence properties, photostability and low toxicity, Feng et al. fabricated a “turn-on” NIR GQD/CoOOH nanoprobe for the two-photon bioimaging of endogenous ascorbic acid in living cells [122]. As shown in Figure 10, the NIR GQDs prepared from polythiophene through the hydrothermal method were coated with a layer of CoOOH nanoflakes, which greatly quenched the NIR fluorescence with quenching efficiency of 97%. In the presence of ascorbic acid in living cells, the NIR fluorescence centered at 660 nm was restored as the CoOOH nanoflakes were reduced to Co^2+^ by ascorbic acid. With this “turn-on” probe, ascorbic acid could be detected in living cells and tissues by both single-photon and two-photon excitation.

**miRNA:** Laurenti et al. introduced a new strategy to exploit GQDs as an intermediate energy level for the energy transfer process to achieve the purpose of sensitizing upconverting nanoparticles (UCNP). Using this strategy, he proposed a method for detecting miRNAs. In the absence of target miRNA, they will be hybridized with the ssDNA functionalized on UCNP, hindering the enhancement of upconversion fluorescence. Conversely, the assembly of GQDs and UCNP would be formed due to the property of sp2 carbons preferring to combine with ssDNA on UCNP through π–π interactions; thus, upconversion emission can be increased conspicuously [65].

Ratnesh et al. synthesized highly fluorescent mGQDs using the green color of mango leaves and constructed an “on–off” nanoprobe based on a fluorescence quenching-recovery mechanism to achieve dual detection of Fe^2+^ ions and cholesterol, as well as molecular logic gate sensing applications [123].

**O_2_^•−^ and •OH:** Superoxide anions (O_2_^•−^) and hydroxyl radicals (•OH), as two common reactive oxygen species (ROS), normally exist at low concentrations; however, their overaccumulation could imply many pathological conditions including inflammation and cancer [124,125,126]. In order to achieve real-time tracing of the ROS level in vivo, fluorescence imaging is often taken into consideration [127,128]. To improve the small Stoke shift and light instability of organic dyes which are usually used in fluorescence imaging, Liu et al. introduced GQDs combined with the organic sensor HydroIR783, which is sensitive to O_2_^•−^ and •OH, that were converted to NIR dye IR_783_, resulting the FRET from GQDs to IR_783_ [129]. Wibowo et al. systematically studied the effects of surface oxygen-containing functional groups and sp^2^ carbon structure on fluorescence properties and antioxidant free radical scavenging ability [130].

**H_2_O_2_:** Wu et al. were inspired by the characteristic of aromatic sp^2^ carbon clusters containing materials with peroxidase-like catalytic activity [131] and designed a label-free colorimetric system for detecting H_2_O_2_ (0.1 mM to 10 mM). Based on this property of GQDs, which have the Stokes shift to NIR fluorescence, H_2_O_2_ could be catalyzed and decomposed. In the meantime, it induced the conversion of ABTS as the chromogenic substrate into green ABTS radicals, resulting in the color of the solution changing from colorless to green. By detecting the absorbance of the solution at 400–700 nm, it can be found that this method is fast and has a better response within PH of 3–5. The reaction rate is proportional to the concentration of GQDs and ABTS [42]. Li et al. synthesized Fe/B-co-doped graphene quantum dots (Fe/B-GQD-HSF) modified with histidine, serine, and folic acid. They utilized its peroxidase-like activity to catalyze the oxidation of TMB by H_2_O_2_, and detected H_2_O_2_ through colorimetric and fluorescence signal changes [132].

**Hg^2+^:** A ratiometric fluorescence sensor has been developed to detect the concentration of Hg^2+^ by comparing the fluorescence intensities of two different wavelengths before and after the sample is added. The core design idea of this sensor is to use electrostatic self-assembly to connect GQDs with blue fluorescence to silica nanospheres coated with CdTe QDs with red fluorescence. The carboxyl and hydroxyl groups modified on the surface of GQDs have a good affinity for Hg^2+^ and facilitate the combination of GQDs with Hg^2+^, resulting in quenched fluorescence of GQDs. Since the fluorescence of CdTe QDs will not be affected, treating it as a control and comparing it with the fluorescence of GQDs, the concentration of Hg^2+^ could be detected sensitively and specifically with satisfactory recovery in real samples [133].

Subsequently, Peng et al. chose 5,10,15,20-tetrakis (1-methyl-4-pyridinio) porphyrin tetra (p-toluene sulfonate) (TMPyP), a natural compound that exhibits high molar absorbance and great fluorescence, for the detection of mercury ions to reduce the potential cytotoxicity caused by heavy metal elements. TMPyP has the property of complexing with small divalent metal ions, but this reaction proceeds very slowly as it struggles to deform the porphyrin ring plane. On the one hand, Hg^2+^ could sit on the top of the porphyrin ring and change its conformation to promote Mn^2+^ to attack complex sites from the rear. On the other hand, the electrostatic and hydrogen bond interaction between NGQDs and TMPyP narrow the distance between Mn^2+^ adsorbed on NGQDs and the TMPyP, thereby increasing the probability of reaction. Once the Mn^2+^ is chelated in the porphyrin ring, it will be oxidized by the oxygen in the water, and the released Hg^2+^ will participate in the next cycle [79]. Peng also proposed an efficiency strategy by utilizing the inner filter effect (IFE). It is unlike the traditional ratiometric fluorescence methods based on intramolecular charge transfer or fluorescence resonance energy transfer (FRET), which require a stringent matching between the fluorophore and quencher for efficient energy/charge transfer processes. It has been proved that the IFE phenomenon is another ideal way for sensors to detect small molecules and is more suited for complex biological applications [134,135]. In this case, it means that, due to an overlap between the emission of GQDs and the excitation of TMPyP, TMPyP would absorb most of the emission from GQDs, resulting in the fluorescence of GQDs being hidden; following a reaction with Mn, the fluorescence of GQDs would recover.

**Glutathione (GSH):** Glutathione is an important molecule containing -SH groups in cells. It plays an extremely important role in the inactivation of oxygen free radicals, organic hydroperoxides and electrophiles. If its level in the body can be monitored in real time, GSH can serve as a risk warning for diseases such as cancer, Alzheimer’s disease [136], human immunodeficiency virus (HIV) [137], cystic fibrosis and heart problems [138]. Manganese dioxide (MnO_2_) nanosheets have a large surface area and excellent light absorption capabilities, which can be used as a trigger structure for “off–on” fluorescence sensors. At the same time, they are sensitive to GSH and can be reduced by GSH to generate harmless Mn^2+^. Therefore, MnO_2_ nanosheets are used as GSH receptors combined with other fluorescent materials to achieve the purpose of detecting GSH [139]. Meng et al. proposed a two-photon nanoprobe for the detection of GSH, which combined GQDs and MnO_2_ nanosheets with both NIR excitation and emission features. The fluorescence was quenched by MnO_2_ and the presence of GSH degraded the MnO_2_ into Mn^2+^, which restored the fluorescence of GQDs for the detection of GSH. Moreover, the consumption of GSH enhanced the PDT effect induced by GQDs [43]. Subsequently, Song et al. improved the above-mentioned nanosheets with irregular and inconsistent sizes and morphologies into nanoflowers, which improved the repeatability of GSH detection in complex biological environments [44].

Overall, red-to-NIR-fluorescent GQD-based biosensors show several advantages for small-molecule detection, including reduced background fluorescence, improved tissue penetration, and good compatibility with cellular imaging. However, their analytical performance varies depending on the target analyte, sensing mechanism, and biological matrix. Ratiometric and “turn-on” fluorescence strategies generally provide better reliability in complex samples because they can reduce interference from probe concentration, excitation fluctuation, and background signals. In contrast, single-signal “turn-off” systems are easier to construct but may be more susceptible to nonspecific quenching and matrix effects. Therefore, the performance of GQD-based biosensors should be evaluated not only by fluorescence response, but also by limit of detection, linear range, response time, selectivity, anti-interference capability, and validation in real biological samples. Future studies should report these parameters more systematically to allow direct comparison among different GQD-based sensing platforms.

### 6.2. Bioimaging In Vitro

A fluorescent probe that can be excellently applied to practical biology imaging studies should meet the following requirements [140,141,142]. Foremost, it should have good biocompatibility and ensure long-term activity in a complex biological environment without photobleaching and blinking. Second, it is necessary to have a high quantum yield to improve the emission efficiency of fluorescence while reducing laser radiation impairment. Third, it must have the ability to be easily modified on surfaces to facilitate connection with other molecules or functional groups to achieve specific purposes, such as targeting tumor cells. Nowadays, more and more fluorescent probes have been put into use, such as GFP protein or certain inorganic quantum dots. On the other hand, they are restricted from certain shortcomings such as being easily photobleached or considerably cytotoxic. GQDs are very promising alternatives. GQDs have many good properties that satisfy the requirements of an excellent fluorescent probe. Different from organic dyes and semiconductive quantum dots, GQDs exhibit high photostability and low cytotoxicity for cellular imaging [62,63]. Moreover, red-to-NIR fluorescence from GQDs would provide deeper tissue penetration and better optical separation from bio-sample autofluorescence. Yan et al. developed a GQD-based fluorescence tumor imaging sensor by planting graphene quantum dots into PEGylated nanoparticles in situ, enabling targeted multimodal molecular imaging with 4× longer blood circulation and ~7–8× higher tumor accumulation compared with free GQDs [143]. In the work of Das, they converted motorcycle exhaust soot into high-quantum-yield GQDs and applied them for sensitive fluorescence detection of ferrocyanide ions and cellular bioimaging [144].

Recently, Gao et al. prepared red-emissive GQDs by coating them with polyethyleneimine (PEI) of different molecular weights. These PEI-coated GQDs showed bright red fluorescence and could be used for imaging human embryonic kidney cell line 293 (HEK-293) and human primary glioblastoma cell line 87 (U-87) cells [67].

**Figure 11:**
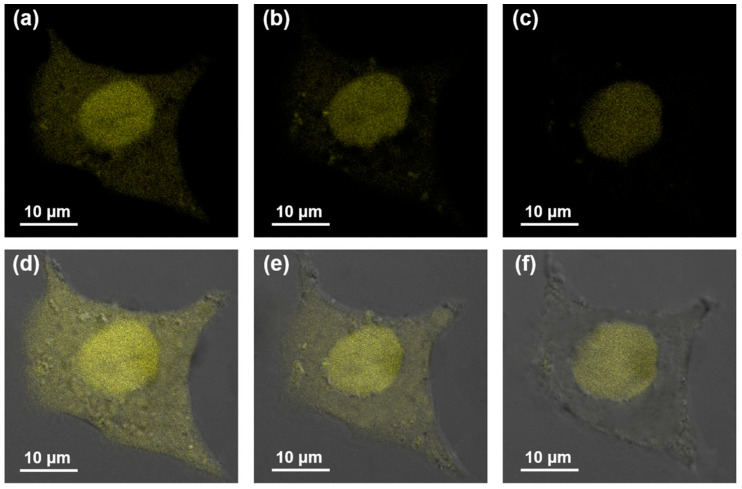
Confocal cell imaging (488 nm laser excitation) of HeLa cells with R-GQDs under different temperatures, namely, 32 °C (**a**,**d**), 37 °C (**b**,**e**), and 42 °C (**c**,**f**). (**a**–**c**) are the fluorescence images of HeLa cells; (**d**–**f**) are the merged (dark field merged with bright field) pictures. Reproduced with permission from Gao et al. [145], *ACS Applied Materials & Interfaces*; published by American Chemical Society, 2020.

For in vitro cellular imaging, GQDs are usually distributed in the cytoplasm and cell membrane without further targeting ligand modification [145] (Figure 11). However, to obtain more site-specific imaging ability, the modification of targeting ligands to different organelles has also been introduced. Wu et al. combined triphenylphosphonium (TPP) and morpholine, which can selectively target mitochondria and lysosomes, with GQDs [115].

While most researchers have focused on exploiting GQDs’ fluorescence for targeting tumor cells, GQDs could be also used as real-time detectors for monitoring GSH [44], temperature [66], and pH [39] in situ to differentiate normal and cancer tissues.

### 6.3. Bioimaging In Vivo

In the field of bioimaging, graphene oxide (GO) has a wide range of applications, such as optical imaging. Non-invasive optical imaging combines the unique properties of visible light and photons to provide comprehensive images of organs and tissues as well as tiny objects such as cells and molecules [146]. Compared with other imaging methods, it has many advantages, including low cost, high sensitivity (10^9^–10^12^ mol/L), no ionizing radiation, real-time imaging, fast acquisition speed, and multiplexing capability. However, this imaging method has a shallow tissue penetration depth (0–2 cm), significant photon scattering in the visible light region (395–600 nm), and considerable background noise due to tissue autofluorescence and light absorption by proteins (257–280 nm), heme groups (absorption peak at 560 nm), and even water (above 900 nm). To address these challenges, researchers have explored near-infrared-window (NIR, 650–900 nm) and second-near-infrared-window (NIR-II, 1000–1700 nm) imaging modalities, which offer advantages such as reduced autofluorescence, reduced tissue scattering, and better in vivo imaging penetration [50,147]. Nunez et al. reported on the bioimaging applications of nanocomposites composed of graphene oxide (GO). They covalently linked boron monoiodide cluster derivatives to GO. In vitro cytotoxicity experiments (lasting 48 h) on HeLa cells showed that the nanocomposites exhibited negligible cytotoxicity with a cell death rate of less than 10%. Furthermore, in vivo experiments showed similar results to in vitro experiments, with *C. elegans* used to demonstrate that the nanocomposites could be ingested by the worm without substantial harm and with extremely low toxicity [148,149].

We investigated the feasibility of GQDs for in vivo NIR bioimaging by subcutaneously and intramuscularly injecting GQDs into nude mice. We found that as the excitation wavelength increases, the emission fluorescence intensity decreases, but the signal-to-noise ratio increases. This suggests that both effects should be taken into consideration when optimizing the excitation and emission bandpass of GQDs in in vivo imaging [42]. Another common method for studying in vivo NIR imaging of GQDs is to mix the prepared GQDs with acrylamide gel and implant the mixture into sacrificed mice so that it is convenient to determine the capability of penetration depth and distribution of GQDs in the organism [68]. Kuo et al. developed a near-infrared two-photon fluorescence sensor based on nitrogen-doped and amino-functionalized graphene quantum dots (amino-N-GQDs), which operate through two-photon excitation-induced photoluminescence, enabling stable excitation-independent NIR fluorescence for sensitive deep-tissue bioimaging detection [150]. Recently, they developed amino-functionalized N-doped graphene quantum dot–polymer nanohybrids that act as efficient contrast probes for NIR-I/II multiphoton bioimaging, enabling deep-tissue (~270 μm), low-energy, and high-contrast imaging of bacteria [151].

Most animal models involve in situ injection or intramuscular injection. This method ignores the metabolism and transportation of the GQDs in the animal and is only suitable for superficial tumors. To realistically simulate the most used method in the clinic, Liu et al. applied intravenous injection to a HeLa tumor-bearing nude mice model. They demonstrated that the developed GQDs accumulated efficiently at the tumor site through the EPR effect. Further investigation of the ex vivo organs of sacrificed mice can also indicate the same conclusion. It implied that GQDs have the potential to target and trace deep tumors [113]. Liu et al. also developed a GQD-HydroIR_783_ nanoprobe to enable real-time monitoring of ROS species and trace inflammation processes in vivo through intraperitoneal injection [129]. Liang et al. used molecular dynamics simulations to study the adsorption behavior of ssDNA and dsDNA on GQDs of different sizes and oxidation levels. The results showed that ssDNA tends to lie flat on the GQD surface, while dsDNA tends to adsorb vertically. Larger GQDs lead to stronger adsorption and greater DNA structural deformation, suggesting potential cytotoxicity [152].

### 6.4. Theragnostic Agents

#### 6.4.1. Fluorescence Imaging + Chemotherapy

In recent years, GQDs have emerged as a fascinating frontier in chemotherapy, presenting a unique combination of nanotechnology and therapeutic innovation. The rationale for using GQDs lies in their ability to overcome the common limitations of traditional chemotherapy for drug delivery. These nanoparticles offer advantages such as improved biocompatibility, enhanced drug stability, and the ability to precisely target cancer cells [153]. However, typically, GQDs emit blue fluorescence, which makes them ineffective in penetrating deep tissues. Therefore, GQDs with short-wavelength fluorescence emission are not suitable for imaging deep tissues in in vivo experiments. When GQDs are used together with near-infrared (NIR) dyes, the problem of fluorescence quenching caused by GQDs is usually encountered because GQDs can quench NIR fluorescence. This problem has greatly hindered the therapeutic progress of GQDs. To overcome this limitation, Ding et al. proposed a therapeutic platform based on GQDs that can simultaneously serve as a carrier and signal generator for anticancer drugs, providing different fluorescent signals at different stages to indicate drug delivery, release, and response [154]. Yao et al. designed GQD–mesoporous silica nanoparticles (MSNs) which can release drugs when the pH value decreases. This is because the hydrogen bonds and electrostatic forces between GQDs and MSNs will be relatively weak and easily destroyed in acid environments [155]. Gomez et al. used nitrogen-doped graphene quantum dots covalently loaded with organotin(IV) metallodrugs to deliver cytotoxic tin compounds into triple-negative breast cancer cells, achieving selective and strong anticancer activity with low IC_50_ values while showing lower toxicity toward normal cells [156].

#### 6.4.2. Fluorescence Imaging + Photodynamic Therapy

On the other hand, GQDs can absorb the energy of 808 nm near-infrared light through conjugated π bonds and convert it into thermal energy, promote rapid heating of the surrounding environment, and generate reactive oxygen species (ROS) to accelerate tumor cell death. Therefore, GQDs are considered as promising photosensitizers [157]. For example, GQDs modified with cRGD can bind to integrins on the surface of cancer cells to achieve targeted therapy against cancer cells. This targeted therapy strategy can improve therapeutic efficacy and reduce damage to normal cells [158]. The drug delivery nanosystem composed of GQDs and magnetic chitosan can use the photothermal properties of GQDs to generate local high temperatures, thereby promoting the thermotherapeutic effect of tumor cells. At the same time, the magnetism of nanoparticles can position and guide tumors through an external magnetic field [159]. Another example is GQDs serving as reducing agents, stabilizers and drug carriers for gold nanosphere clusters. GQD–gold nanosphere clusters can not only be used for photoacoustic imaging (PAI) and computed tomography (CT) imaging, but also, in the case of GQD-delivered doxorubicin, can be used for chemotherapy–photothermal treatment by controlling drug release in the heated and acidic environment of the tumor [160]. Kuo et al. used nitrogen-doped and amino-functionalized graphene quantum dots as two-photon photodynamic therapy (PDT) photosensitizers, where near-infrared two-photon excitation generates reactive oxygen species (ROS), resulting in efficient killing of multidrug-resistant cancer cells [150].

**Figure 12:**
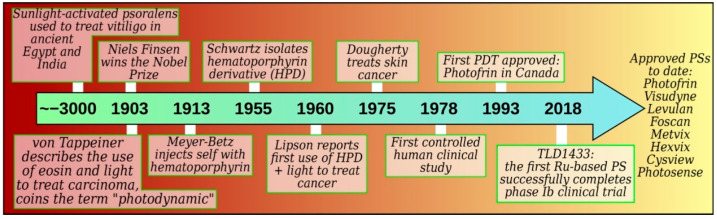
Important events in the history of PDT. Reproduced with permission from Monro et al. [161], *Chemical Reviews*; published by American Chemical Society, 2018.

Photodynamic therapy (PDT) was first proposed around the turn of the 20th century (Figure 12). It is a form of phototherapy using photosensitizers, which, under excited conditions, would catalyze the photosensitized oxidation reactions with oxygen to damage or destroy unwanted living tissue (phototoxicity) [161]. Milenkovic et al. functionalized graphene quantum dots with curcumin to construct a photosensitizer that enhances singlet oxygen generation for photodynamic therapy [162].

**Figure 13:**
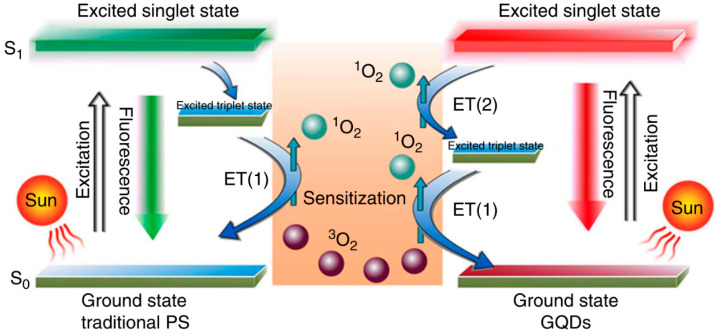
The ^1^O_2_ generation mechanisms of traditional PDT agents (**left**) and GQDs (**right**). The arrows indicate excitation, fluorescence emission, and energy-transfer (ET) processes that sensitize triplet oxygen (^3^O_2_) to singlet oxygen (^1^O_2_). Reproduced with permission from Ge et al. [47], *Nature Communications*; published by Springer Nature, 2014.

Due to its minimal invasiveness, few side effects, reduced resistance, and negligible cytotoxicity, PDT has been approved for clinical treatment in cases such as malignant cancers and prostate cancer [163]. However, the application of current PDT agents is impeded owing to their poor water dispersibility, photostability, low penetration, cytotoxicity, low ROS-generation efficiency, and low quantum yield [164]. Ge et al. proposed to use GQDs as a PDT reagent, which not only has an absorption peak in the infrared region and improves the problem of poor penetration of the PDT reagent, but also has good biocompatibility, high quantum yield and excellent killing effect of HeLa cells both in vitro and in vivo. The mechanism of its high quantum yield is also discussed. This may be attributed to a new ^1^O_2_-generating mechanism called multistate sensitization (MSS) in which the energy gap between excited singlet states (S1) and the excited triplet states (T) is larger than the formation energy of ^1^O_2_ compared with the traditional PDT reagent so that oxygen can be twice-excited and converted into ^1^O_2_ (Figure 13). This suggests that GQDs could be a promising PDT reagent [47].

#### 6.4.3. Fluorescence Imaging + Photothermal Therapy

Unlike photodynamic therapy, photothermal therapy (PTT) does not require oxygen to achieve a therapeutic purpose, which allows it to overcome hypoxia in the tumor microenvironment. It employs electromagnetic radiation (generally NIR wavelengths) and has been increasingly regarded as a suitable modality to treat a wide range of medical conditions, including cancer, precisely and non-invasively. The principal mechanism of PTT is excitation of sensitizers that have high photothermal conversion efficiency; they then release vibrational energy (heat) at the target area, where the rise in temperature can increase the permeability of the cell membrane to inhibit normal metabolism [165].

Photothermal conversion agents (PTCAs) play a vital role in PTT, including polypyrrole nanoparticles (NPs), polyaniline NPs, and metal NPs [166,167,168]. Due to the potential cytotoxicity and immunogenic, most of these PTCAs are difficult to apply clinically [169]. First, researchers would like to take advantage of GQDs as carriers to transport the organic NIR PTCA IR_780_ iodide (IR_780_) to overcome its poor photostability, as well as to solve the hydrosolubility problem through modification of folic acid (FA) on GQD surfaces via π–π stacking interactions for targeting the tumor. Upon 808 nm laser irradiation, it shows the capability to induce hyperthermia and tumor cell apoptosis [75].

However, organic PTCAs also face many challenges, such as short fluorescence lifetime, poor photobleaching resistance, and photostability. GQDs not only can perfectly overcome these defects but also have the advantages of large Stokes displacement, wide excitation spectrum, and narrow emission spectrum. Xuan et al. designed nitrogen-doped GQDs with low toxicity, high NIR photothermal conversion efficiency, and high quantum yield. They also proposed the first application of GQDs in photoacoustic imaging (PAI) [45]. In PAI, non-ionizing laser energy is absorbed by biological tissue, and some of the delivered energy produces thermal expansion, leading to transient wideband (i.e., MHz) ultrasonic waves that contain the characteristic information of tissue and are detected by ultrasonic transducers. Afterward, the signal would be reconstructed and analyzed to generate distribution images. Not only could PAI avoid light scattering to achieve deep tissue, but it could also realize high-contrast optical imaging, which is an extremely promising application for early diagnosis and efficacy monitoring [170].

It was observed by Wang et al. that the photothermal effect of GQDs can be employed not only for the traditional NIR (750–900 nm) region but also for the second NIR region (NIR-II, 1000–1700 nm) [46]. As the imaging wavelength is longer, the light scattering and autofluorescence of tissue will be reduced, so compared to imaging in the visible light region (400–750 nm) and the traditional NIR zone, fluorescence technology in the NIR-II zone shows better performance in avoiding background interference and has been increasingly applied to the field of PTT as well [171]. This work also displayed the potential of GQDs for evaluating the optical and therapeutic properties of tumors in a mouse model.

#### 6.4.4. Fluorescence Imaging + Gene Therapy

There are two main ways for nanomaterials to enter tumor tissue. One is passive targeting, which is mainly attributed to the tumor’s enhanced permeability and retention (EPR) effect. In the tumor microenvironment, the vascular endothelium grows too fast to be closely connected, resulting in abnormal molecular and fluid transport dynamics, which benefits the enrichment of macromolecular drugs or nanoparticles. The approach other is active targeting, mainly by attaching small molecules that can specifically recognize tumors to nanomaterials, including functional small molecules, peptides, antibodies, and oligonucleotides [172,173,174,175].

AS1411 is a nucleic acid sequence that forms a quadruplex structure due to the large amount of guanine [176]. It is targeted to Nucleolin (C_23_) receptors expressed on cancer cell membranes. After it enters cells, it can mediate a series of pro-apoptotic reactions and inhibit tumor cell growth [177]. Meanwhile, it shows excellent stability in bio-samples due to its resistance to nuclease degradation. Wang et al. took advantage of this feature to cross-link AS1411 through the EDC/NHS reaction on GQDs that generate photothermal effects under 808 nm excitation, thereby not only enabling GQDs to specifically identify tumor cells but also to enhance the ability to kill tumors [178].

#### 6.4.5. Multi-Modality Therapy

Due to the rapid spread of antibacterial resistance, more and more attention has been paid to developing alternative antibacterial materials. Artificial enzymes, which are synthesized by nanotechnology, show promising prospects of application, inducing irreversible damage toward bacteria. Chen S et al. first attempted to combine GQDs with Ag nanoparticles, showing peroxidase-mimicking and oxidase-mimicking activity, which can both convert H_2_O_2_ into •OH and generate reactive oxygen species (ROS) to inhibit growth of Gram-negative, Gram-positive and drug-resistant bacteria. This work demonstrated that GQDs could open new opportunities for treating other significant diseases, which will expand the applications of GQDs in the biomedical field [83].

Redox homeostatic balance is essential to cell performance, including gene expression. Aggressive cancer cells have the tendency to increase reactive oxygen species (ROS) to enhance the level of gene expression [179]. Biological redox therapy (BRT) is a new attempt that disrupts redox homeostasis by promoting oxidant accumulation, thereby inducing oxidative stress and tumor cell death [180]. Yang et al. designed a multifunctional therapy agent using GQDs for PTT and a carrier loading Gadolinium (III) texaphyrin (Gd-TP) for BRT and lutetium (III) texaphyrin (Lu-TP) for PDT. Upon NIR irradiation, GQDs generate an amount of heat that both promotes endocytosis of drugs and impedes normal physiological activities. Subsequently, fluorescence would recover after drug release, which could be used as a means of assessment by MRI and fluorescence imaging [181]. Kurniawan et al. developed a plasma-engineered GQD hydrogel as a pH-responsive nanocarrier for controlled delivery of the anticancer drug doxorubicin (DOX) to cancer cells [182]. Das et al. synthesized GQDs, N-GQDs, and S-GQDs, studied their interactions with the anticancer drug methotrexate (MTX), and found that GQDs can enhance the killing effect of MTX on HeLa cancer cells, suggesting their potential application in a cancer therapy nanoplatform [183]. Poursadegh et al. prepared graphene quantum dots via pyrolysis and formed magnetic GQDs with Fe_3_O_4_. Simultaneously, they grew MIL-88(Fe) MOF in situ and incorporated it into PVC to prepare a composite film, thus achieving a highly efficient microwave-absorbing material [184]. Milenkovic et al. synthesized Au nanoparticle-decorated graphene quantum dots (GQD-AuNPs) via γ-irradiation and demonstrated that the composites generate singlet oxygen under blue light and exhibit biocompatible antibacterial activity, particularly effective against MRSA [185]. In Rahardja’s work, they fabricated GQD-crosslinked hydrogels that enable high-capacity anticancer drug delivery (doxorubicin) and efficient adsorption of toxic dyes for environmental remediation [186]. Godwin et al. synthesized GQDs from agricultural waste (groundnut and Medicago sativa oil cakes) via a hydrothermal method and demonstrated their strong photoluminescence, good biocompatibility, and promising biomedical activities including antioxidant, anti-inflammatory, and antibacterial effects [187]. In the work of Kadyan et al., red-fluorescent GQDs exhibited strong antioxidant activity and anti-diabetic potential through inhibition of α-amylase and α-glucosidase [188].

Nitric oxide (NO) is an important active molecule in living creatures involved in mediating various cell activities, including vascular growth, smooth muscle relaxation, immune response, apoptosis, and synaptic information transmission. High concentrations of NO have been confirmed to kill tumor cells [189,190]. However, NO is unstable and easily degraded by oxygen, metal enzymes, and other substances in a complex biological environment. How to increase the NO concentration inside tumor tissue has always been under discussion. Li et al. designed a treatment platform that uses GQDs as a carrier to connect low-toxicity, high-stability and light-excitable NO (donor) (Ru-NO) to a galactose derivative (Gal) which can target receptors on the surface of liver cell membranes. It not only meets the requirement of specifically targeting the tumor but also can control the release of NO and achieve the purpose of PTT by 808 nm NIR light [191].

Taken together, GQD-based therapeutic platforms are attractive because they can integrate fluorescence imaging with chemotherapy, PDT, PTT, gene therapy, antibacterial therapy, or gas therapy in a single nanosystem. Compared with single-modality treatment, these combined strategies may improve therapeutic efficiency through complementary mechanisms, including controlled drug release, ROS generation, photothermal heating, redox regulation, and NO delivery. However, true therapeutic synergy should be distinguished from a simple additive effect, and quantitative evaluation using combination index, tumor inhibition ratio, survival analysis, or dose-reduction effect remains limited in many studies. In addition, combined PTT/PDT or chemo-photothermal systems may introduce potential side effects, such as local overheating, oxidative stress in normal tissues, nonspecific drug release, dark toxicity, phototoxicity, and long-term accumulation of nanomaterials. Therefore, future studies should provide more systematic comparisons of therapeutic efficacy, synergistic effect, biodistribution, clearance, and long-term biosafety before these platforms can be further translated toward biomedical applications.

## 7. Perspective

The emergence of red-to-NIR-fluorescent GQDs marks a transformative advancement in nanotechnology, particularly within biomedical sciences. As a subclass of C-dots (CDs), GQDs possess similar biocompatibility and biosafety while retaining tunable photoluminescence, excellent stability, and environmentally friendly characteristics, making them attractive candidates for biomedical applications. Unlike traditional GQDs, which typically emit fluorescence in the ultraviolet or visible spectrum, red-to-NIR GQDs address the challenges of high autofluorescence and limited tissue penetration. Their emission in the NIR region ensures superior imaging depth and clarity, making them highly advantageous for various biomedical applications.

In bioimaging, red-to-NIR GQDs have demonstrated their ability to overcome conventional challenges of poor signal-to-noise ratios and shallow penetration depths, providing an efficient platform for both in vitro and in vivo imaging. Their photostability and low toxicity enhance their suitability for clinical imaging applications, while surface modifications allow precise targeting of cellular and subcellular structures, enabling the study of complex biological processes with high resolution. Similarly, in biosensing, GQDs exhibit exceptional sensitivity in detecting biomolecules such as miRNA and reactive oxygen species (ROS), facilitating early disease diagnosis and real-time monitoring. These capabilities are particularly significant in addressing critical healthcare challenges, such as cancer detection and metabolic disorder management.

Beyond diagnostics, red-to-NIR GQDs also hold immense promise as theragnostic agents. Their dual functionality, combining imaging and therapy, enables precise, non-invasive treatments such as photothermal and photodynamic therapies, which are highly effective in targeting cancer cells while minimizing collateral damage to healthy tissues. Furthermore, the ease of synthesis and surface modification of GQDs supports the integration of additional therapeutic modalities, such as drug delivery and gene therapy, expanding their versatility and impact in personalized medicine.

Despite these advancements, several challenges must be addressed to fully harness the potential of red-to-NIR-fluorescent GQDs, including GMP-compliant manufacturing, batch-to-batch consistency, standardized quality control (e.g., size distribution, polydispersity index, and quantum yield), regulatory approval pathways (e.g., FDA and EMA), and benchmarking against conventional organic fluorophores and semiconductor quantum dots. Furthermore, systematic investigations of their long-term biosafety, including toxicity, biocompatibility, metabolism, immunogenicity, and organ accumulation, are essential to facilitate their clinical translation. Recently, machine-learning-assisted GQD design has been greatly investigated to synthesize predictable properties of GQDs, and might be able to adjust the photoluminescence properties, such as excitation/emission wavelengths, fluorescence lifetime, and quantum yield [192]. Furthermore, machine learning-assisted methods have started to be used for metal detection based on GQDs [193].

Looking ahead, the future of red-to-NIR-fluorescent GQDs lies in interdisciplinary collaboration between materials science, chemistry, and biomedical research. By addressing the existing limitations and advancing our understanding of these materials, we can unlock new frontiers in healthcare diagnostics, therapy, and imaging. Red-to-NIR-fluorescent GQDs have the potential to revolutionize nanomedicine, contributing to precision medicine and improving global healthcare outcomes. With sustained efforts in research and development, these innovative materials are poised to play a pivotal role in the next generation of biomedical technologies.

## Figures and Tables

**Table 1:** Overview of bottom-up methods for preparing red-to-NIR-fluorescent GQDs.

Precursors	Synthetic Method	Emission Wavelength	Quantum Yield (%)	Application	Size (nm)	Ref.
Poly (3-alkylthiophenes)	Hydrothermal treatment 170 °C for 20 h	~700 nm	/	Cell imaging in C57BL/6 mice dendritic cells and human adipose-derived stem cell cytoplasm	3.1	[37]
Glucosamine-HCl	Microwave treatment (at 450 W) with or without thiourea.	~800 nm or ~850 nm	50–60% or 10–20%	/	5.50 or 3.90	[38]
Glucosamine-HCl with different dopant precursors (sulfur thiourea or benezeneboronic acid)	Microwave treatment (at 450 W) 40 min	~870 nm or ~890 nm	22–60%	Cell imaging in HEK-293, HeLa and MCF-7 cell cytoplasm and pH sensor	3–5	[39]
Glucosamine-HCl	Microwave treatment for 60 min at 1350 W, then ozone treatment at 10% (0.3 g·L^−1^) of maximum ozone level (3 g·L^−1^) for 0–65 min	800~825 nm	/	Solar cell applications	6	[40]
Glucose	Adding aqueous ammonia and heating in a microwave oven at 280 W for 1, 3, 5, 7 and 9 min.	~917 nm	6.8–11.3%	/	1.7–5.8	[41]
L-glutamic acid	Heat to 210 °C 45 s then add 10.0 mL water for 30 min	815 nm	54.5%	Cell imaging in MH-S cell cytoplasm and in vivo detection of H_2_O_2_	4.66 ± 1.24	[42]
Polythiophene (PT2)	Hydrothermal treatment 170 °C for 24 h	665 nm	/	Detection of GSH	2–5	[43,44]
Citric acid and urea	Heat to 160 °C for 12 h	830 nm	2.49%	Photothermal therapy	5	[45]
3-aminophenylboronic acid monohydrate (APBA)	Sonicate for 30 min in acetone then add H_2_O_2_ (30%). Ultrasonicate for 10 min, then heat to 230 °C. for 24 h	1000 nm	/	Photothermal therapy	∼4.7	[46]
Polythiophene (PT2).	Disperse in NaOH solution, ultrasonicate for 30 min, heat at 170 °C for 24 h.	680 nm	5.4%	Photodynamic therapy	2–6	[47]

**Table 2:** Overview of top-down methods for preparing red-to-NIR-fluorescent GQDs.

Precursors	Synthetic Method	Emission Wavelength	Quantum Yield (%)	Application	Size (nm)	Ref.
Graphite	Electrochemical exfoliation of graphite by K_2_S_2_O_8_ solution	610 nm	/	Cell imaging in HeLa cell membrane and cytoplasm	3 nm	[62]
Activated carbon	Oxidation with nitric acid 140 °C for 3 h	600 nm	18%	Cell imaging in CHO-K1 cell cytoplasm	3.3–12 nm	[63]
GO sheets	Incubate with H_2_O_2_ and thiourea at 120 °C for 10 min	630 nm	1%	/	5–10 nm	[64]
Graphite	Hydrothermal method (treat with HF and KMnO_4_)	630 nm	/	Detection for miRNA	4.3 ± 0.8 nm	[65]
Mango leaves	Dip in absolute ethanol for 4 h, then centrifuge at 8000 rpm for 10 min; collect the extract then evaporate the ethanol. The residuals are mixed with water and heated under 900 W microwave oven for 5 min	680 nm	/	Cell imaging in L929 cell cytoplasm and temperature sensor	2–8 nm	[66]
VCX-72 carbon black	Refluxed in HNO_3_ for 24 h, treated by ultrasonication for 10 min at 950 W and then centrifuged (8000 rpm) for 10 min	622 nm	/	Cell imaging in U-87 cell cytoplasm	57.31 ± 8.90 nm	[67]
Highly oriented pyrolytic graphite (HOPG) plate	Immersed in polyethylene glycol and then ablated by a focused nanosecond pulsed laser 30 min	600 nm	47.16%	In vivo imaging	2–10 nm	[68]

**Table 3:** The adsorption affinity between different GQD oxidation level and ssDNA, phospholipids, and protein-like polymers.

Biopolymer Adsorbate Type	Biopolymer Sequence	GQD Adsorbent Type
ssDNA	(GT)_15_	No-ox-GQD > Low-ox-GQD
	T_30_	No-ox-GQD > Low-ox-GQD
	A_30_	No-ox-GQD > Low-ox-GQD
	G_20_	No-ox-GQD > Low-ox-GQD
	C_30_	No-ox-GQD
Phospholipid	14:0 PE-DTPA	Low-ox-GQD
Peptoid	(Nae-Npe)_9_-(Nce-Npe)_9_	No-ox-GQD
	(Nce-Npe)_9_	None

## Data Availability

Data are contained within the article.

## References

[1] Bacon M., Bradley S.J., Nann T. (2014). Graphene Quantum Dots. Part. Part. Syst. Charact..

[2] Ponomarenko L.A., Schedin F., Katsnelson M.I., Yang R., Hill E.W., Novoselov K.S., Geim A.K. (2008). Chaotic Dirac Billiard in Graphene Quantum Dots. Science.

[3] Ghanbarlou S., Kahforoushan D., Abdollahi H., Zarrintaj P., Alomar A., Villanueva C., Davachi S.M. (2026). Corrigendum to “Advances in quantum dot-based fluorescence sensors for environmental and biomedical detection” [Talanta 294 (2025) 128176]. Talanta.

[4] Geim A.K., Novoselov K.S. (2010). The rise of graphene. Nanoscience and Technology: A Collection of Reviews from Nature Journals.

[5] Li L., Wu G., Yang G., Peng J., Zhao J., Zhu J.-J. (2013). Focusing on luminescent graphene quantum dots: Current status and future perspectives. Nanoscale.

[6] Chong Y., Ma Y., Shen H., Tu X., Zhou X., Xu J., Dai J., Fan S., Zhang Z. (2014). The in vitro and in vivo toxicity of graphene quantum dots. Biomaterials.

[7] Sadana S., Rajamohan N., Manivasagan R., Raut N., Paramasivam S., Gatto G., Kumar A. (2025). Graphene-based materials for photocatalytic and environmental sensing applications. Results Eng..

[8] Fang Y., Wu W.F., Zhao Y., Liu H.Q., Li Z.D., Li X.B., Zhang M.W., Qin Y.A. (2023). Transcriptomic and metabolomic investigation of molecular inactivation mechanisms in Escherichia coli triggered by graphene quantum dots. Chemosphere.

[9] Tian P., Tang L., Teng K.S., Lau S.P. (2018). Graphene quantum dots from chemistry to applications. Mater. Today Chem..

[10] Wang L.Z., Zhang Y., Li L.H., Geng X.Z., Dou D.D., Yu L., Jing H.Y., Fan Y.B. (2023). Graphdiyne oxide elicits a minor foreign-body response and generates quantum dots due to fast degradation. J. Hazard. Mater..

[11] Frangioni J.V. (2003). In vivo near-infrared fluorescence imaging. Curr. Opin. Chem. Biol..

[12] Allen M.J., Tung V.C., Kaner R.B. (2010). Honeycomb Carbon: A Review of Graphene. Chem. Rev..

[13] Kadyan P., Malik R., Bhatia S., Al Harrasi A., Mohan S., Yadav M., Dalal S., Ramniwas S., Kumar Kataria S., Arasu T. (2023). Comprehensive Review on Synthesis, Applications, and Challenges of Graphene Quantum Dots (GQDs). J. Nanomater..

[14] Qin W., Wang M., Li Y., Li L., Abbas K., Li Z., Tedesco A.C., Bi H. (2024). Recent advances in red-emissive carbon dots and their biomedical applications. Mater. Chem. Front..

[15] Baker S.N., Baker G.A. (2010). Luminescent carbon nanodots: Emergent nanolights. Angew. Chem. Int. Ed..

[16] Shen J., Zhu Y., Yang X., Li C. (2012). Graphene quantum dots: Emergent nanolights for bioimaging, sensors, catalysis and photovoltaic devices. Chem. Commun..

[17] Zhao J., Chen G., Zhu L., Li G. (2011). Graphene quantum dots-based platform for the fabrication of electrochemical biosensors. Electrochem. Commun..

[18] Zhao H., Chang Y., Liu M., Gao S., Yu H., Quan X. (2013). A universal immunosensing strategy based on regulation of the interaction between graphene and graphene quantum dots. Chem. Commun..

[19] Liu J.-J., Zhang X.-L., Cong Z.-X., Chen Z.-T., Yang H.-H., Chen G.-N. (2013). Glutathione-functionalized graphene quantum dots as selective fluorescent probes for phosphate-containing metabolites. Nanoscale.

[20] Chakraborti H., Sinha S., Ghosh S., Pal S.K. (2013). Interfacing water soluble nanomaterials with fluorescence chemosensing: Graphene quantum dot to detect Hg^2+^ in 100% aqueous solution. Mater. Lett..

[21] Sun H., Gao N., Wu L., Ren J., Wei W., Qu X. (2013). Highly Photoluminescent Amino-Functionalized Graphene Quantum Dots Used for Sensing Copper Ions. Chem. A Eur. J..

[22] Li X., Zhu S., Xu B., Ma K., Zhang J., Yang B., Tian W. (2013). Self-assembled graphene quantum dots induced by cytochrome c: A novel biosensor for trypsin with remarkable fluorescence enhancement. Nanoscale.

[23] Razmi H., Mohammad-Rezaei R. (2013). Graphene quantum dots as a new substrate for immobilization and direct electrochemistry of glucose oxidase: Application to sensitive glucose determination. Biosens. Bioelectron..

[24] Wang Y., Zhang L., Liang R.-P., Bai J.-M., Qiu J.-D. (2013). Using graphene quantum dots as photoluminescent probes for protein kinase sensing. Anal. Chem..

[25] Zhou X., Zhang Y., Wang C., Wu X., Yang Y., Zheng B., Wu H., Guo S., Zhang J. (2012). Photo-Fenton reaction of graphene oxide: A new strategy to prepare graphene quantum dots for DNA cleavage. ACS Nano.

[26] Chen X., Zhou X., Han T., Wu J., Zhang J., Guo S. (2013). Stabilization and induction of oligonucleotide i-motif structure via graphene quantum dots. ACS Nano.

[27] Jing Y., Zhu Y., Yang X., Shen J., Li C. (2011). Ultrasound-triggered smart drug release from multifunctional core−shell capsules one-step fabricated by coaxial electrospray method. Langmuir.

[28] Zheng X.T., He H.L., Li C.M. (2013). Multifunctional graphene quantum dots-conjugated titanate nanoflowers for fluorescence-trackable targeted drug delivery. RSC Adv..

[29] Jiang F., Chen D., Li R., Wang Y., Zhang G., Li S., Zheng J., Huang N., Gu Y., Wang C. (2013). Eco-friendly synthesis of size-controllable amine-functionalized graphene quantum dots with antimycoplasma properties. Nanoscale.

[30] Markovic Z.M., Ristic B.Z., Arsikin K.M., Klisic D.G., Harhaji-Trajkovic L.M., Todorovic-Markovic B.M., Kepic D.P., Kravic-Stevovic T.K., Jovanovic S.P., Milenkovic M.M. (2012). Graphene quantum dots as autophagy-inducing photodynamic agents. Biomaterials.

[31] Zhu S., Zhang J., Qiao C., Tang S., Li Y., Yuan W., Li B., Tian L., Liu F., Hu R. (2011). Strongly green-photoluminescent graphene quantum dots for bioimaging applications. Chem. Commun..

[32] Wu C., Wang C., Han T., Zhou X., Guo S., Zhang J. (2013). Insight into the cellular internalization and cytotoxicity of graphene quantum dots. Adv. Healthc. Mater..

[33] Nurunnabi M., Khatun Z., Huh K.M., Park S.Y., Lee D.Y., Cho K.J., Lee Y.-k. (2013). In vivo biodistribution and toxicology of carboxylated graphene quantum dots. ACS Nano.

[34] Zhu S., Zhang J., Tang S., Qiao C., Wang L., Wang H., Liu X., Li B., Li Y., Yu W. (2012). Surface chemistry routes to modulate the photoluminescence of graphene quantum dots: From fluorescence mechanism to up-conversion bioimaging applications. Adv. Funct. Mater..

[35] Liu Q., Guo B., Rao Z., Zhang B., Gong J.R. (2013). Strong two-photon-induced fluorescence from photostable, biocompatible nitrogen-doped graphene quantum dots for cellular and deep-tissue imaging. Nano Lett..

[36] Nurunnabi M., Khatun Z., Reeck G.R., Lee D.Y., Lee Y.-k. (2013). Near infra-red photoluminescent graphene nanoparticles greatly expand their use in noninvasive biomedical imaging. Chem. Commun..

[37] Huang D., Zhou H., Wu Y., Wang T., Sun L., Gao P., Sun Y., Huang H., Zhou G., Hu J. (2019). Bottom-up synthesis and structural design strategy for graphene quantum dots with tunable emission to the near infrared region. Carbon.

[38] Hasan M.T., Gonzalez-Rodriguez R., Ryan C., Faerber N., Coffer J.L., Naumov A.V. (2018). Photo-and Electroluminescence from Nitrogen-Doped and Nitrogen–Sulfur Codoped Graphene Quantum Dots. Adv. Funct. Mater..

[39] Campbell E., Hasan M.T., Gonzalez Rodriguez R., Akkaraju G.R., Naumov A.V. (2019). Doped Graphene Quantum Dots for Intracellular Multicolor Imaging and Cancer Detection. ACS Biomater. Sci. Eng..

[40] Hasan M.T., Gonzalez-Rodriguez R., Ryan C., Pota K., Green K., Coffer J.L., Naumov A.V. (2019). Nitrogen-doped graphene quantum dots: Optical properties modification and photovoltaic applications. Nano Res..

[41] Tang L., Ji R., Li X., Bai G., Liu C.P., Hao J., Lin J., Jiang H., Teng K.S., Yang Z. (2014). Deep ultraviolet to near-infrared emission and photoresponse in layered N-doped graphene quantum dots. ACS Nano.

[42] Wu X., Tian F., Wang W., Chen J., Wu M., Zhao J.X. (2013). Fabrication of highly fluorescent graphene quantum dots using L-glutamic acid for in vitro/in vivo imaging and sensing. J. Mater. Chem. C.

[43] Meng H.M., Zhao D., Li N., Chang J. (2018). A graphene quantum dot-based multifunctional two-photon nanoprobe for the detection and imaging of intracellular glutathione and enhanced photodynamic therapy. Analyst.

[44] Song Z.L., Dai X., Li M., Teng H., Song Z., Xie D., Luo X. (2018). Biodegradable nanoprobe based on MnO_2_ nanoflowers and graphene quantum dots for near infrared fluorescence imaging of glutathione in living cells. Mikrochim. Acta.

[45] Xuan Y., Zhang R.-Y., Zhang X.-S., An J., Cheng K., Li C., Hou X.-L., Zhao Y.-D. (2018). Targeting N-doped graphene quantum dot with high photothermal conversion efficiency for dual-mode imaging and therapy in vitro. Nanotechnology.

[46] Wang H., Mu Q., Wang K., Revia R.A., Yen C., Gu X., Tian B., Liu J., Zhang M. (2019). Nitrogen and Boron Dual-Doped Graphene Quantum Dots for Near-Infrared Second Window Imaging and Photothermal Therapy. Appl. Mater. Today.

[47] Ge J., Lan M., Zhou B., Liu W., Guo L., Wang H., Jia Q., Niu G., Huang X., Zhou H. (2014). A graphene quantum dot photodynamic therapy agent with high singlet oxygen generation. Nat. Commun..

[48] Pan D., Zhang J., Li Z., Wu M. (2010). Hydrothermal route for cutting graphene sheets into blue-luminescent graphene quantum dots. Adv. Mater..

[49] Zulhanip A.Z., Hadis N.S.M., Radzol A.R.M., Zulkifli Z., Rosli A.D. (2026). Synthesis of graphene quantum dots via one step hydrothermal cutting: The synergistic effect of graphene oxide and sodium hydroxide. Int. J. Nanoelectron. Mater..

[50] La Ferla B., Vercelli B. (2023). Red-Emitting Carbon Quantum Dots for Biomedical Applications: Synthesis and Purification Issues of the Hydrothermal Approach. Nanomaterials.

[51] Kansara V., Patel M. (2024). Modulating the properties of graphene quantum dots by heteroatom doping for biomedical applications. Colloids Surf. A-Physicochem. Eng. Asp..

[52] Yu G.J., Yoo J.H., Bin Kwon S., Yoo H.C., Lee U.Y., Kang B.G., Kang B.K., Yoon D.H. (2025). Green synthesis of nitrogen-doped graphene quantum dots by recycling waste graphite and impact of oxygen adsorption on enhancing photoluminescence. Surf. Interfaces.

[53] Zhu H., Wang X., Li Y., Wang Z., Yang F., Yang X. (2009). Microwave synthesis of fluorescent carbon nanoparticles with electrochemiluminescence properties. Chem. Commun..

[54] Le K.V., Nguyen H., Le T.P., Le T.V., Phan N.V.G., Trinh K.T.L., Vu T.H., Phan B.T., Nguyen L.T.M., Pham N.K. (2026). Sustainable synthesis of graphene quantum dots with antioxidant, anti-inflammatory and photocatalytic efficiency from biowaste and synergistic antibacterial activity of CuO@GQDs composite. Colloids Surf. A-Physicochem. Eng. Asp..

[55] Kadyan P., Kumar M., Tufail A., Ragusa A., Kataria S.K., Dubey A. (2025). Microwave-assisted green synthesis of fluorescent graphene quantum dots (GQDs) using Azadirachta indica leaves: Enhanced synergistic action of antioxidant and antimicrobial effects and unveiling computational insights. Mater. Adv..

[56] Roch R., Deschanels X., Singaravelu C.M., André N., Rey C., Causse J. (2024). Evidence of the contribution of molecular fluorophores to the luminescence of carbon entities formed by solvothermal treatment of trinitropyrene. RSC Adv..

[57] Mohamed K.M., Winston A., Akash K., Sagayaraj P., Rajeshkumar S., Ravindhran R., Jayanthi S.A., Vijaya J.J. (2024). Novel green synthesis of Value-Added graphene quantum dots from bagasse and pith for biological applications. Biocatal. Agric. Biotechnol..

[58] Tajik S., Dourandish Z., Zhang K., Beitollahi H., Van Le Q., Jang H.W., Shokouhimehr M. (2020). Carbon and graphene quantum dots: A review on syntheses, characterization, biological and sensing applications for neurotransmitter determination. RSC Adv..

[59] Kundu S., Yadav R.M., Narayanan T., Shelke M.V., Vajtai R., Ajayan P.M., Pillai V.K. (2015). Synthesis of N, F and S co-doped graphene quantum dots. Nanoscale.

[60] Maiti S., Kundu S., Roy C.N., Das T.K., Saha A. (2017). Synthesis of excitation independent highly luminescent graphene quantum dots through perchloric acid oxidation. Langmuir.

[61] Li Y., Zhao Y., Cheng H., Hu Y., Shi G., Dai L., Qu L. (2012). Nitrogen-doped graphene quantum dots with oxygen-rich functional groups. J. Am. Chem. Soc..

[62] Tan X., Li Y., Li X., Zhou S., Fan L., Yang S. (2015). Electrochemical synthesis of small-sized red fluorescent graphene quantum dots as a bioimaging platform. Chem. Commun..

[63] Shao T., Wang G., An X., Zhuo S., Xia Y., Zhu C. (2014). A reformative oxidation strategy using high concentration nitric acid for enhancing the emission performance of graphene quantum dots. RSC Adv..

[64] Ke C.-C., Yang Y.-C., Tseng W.-L. (2016). Synthesis of Blue-, Green-, Yellow-, and Red-Emitting Graphene-Quantum-Dot-Based Nanomaterials with Excitation-Independent Emission. Part. Syst. Charact..

[65] Laurenti M., Paez-Perez M., Algarra M., Alonso-Cristobal P., Lopez-Cabarcos E., Mendez-Gonzalez D., Rubio-Retama J. (2016). Enhancement of the Upconversion Emission by Visible-to-Near-Infrared Fluorescent Graphene Quantum Dots for miRNA Detection. ACS Appl. Mater. Interfaces.

[66] Kumawat M.K., Thakur M., Gurung R.B., Srivastava R. (2017). Graphene Quantum Dots from *Mangifera indica*: Application in Near-Infrared Bioimaging and Intracellular Nanothermometry. ACS Sustain. Chem. Eng..

[67] Gao T., Wang X., Yang L.Y., He H., Ba X.X., Zhao J., Jiang F.L., Liu Y. (2017). Red, Yellow, and Blue Luminescence by Graphene Quantum Dots: Syntheses, Mechanism, and Cellular Imaging. ACS Appl. Mater. Interfaces.

[68] Narasimhan A.K., Lakshmi B S., Santra T.S., Rao M.S.R., Krishnamurthi G. (2017). Oxygenated graphene quantum dots (GQDs) synthesized using laser ablation for long-term real-time tracking and imaging. RSC Adv..

[69] Ahirwar S., Mallick S., Bahadur D. (2017). Electrochemical Method to Prepare Graphene Quantum Dots and Graphene Oxide Quantum Dots. ACS Omega.

[70] Zhang C.Y., Cai Z.Z., Chu K., Shiu W.T., Hu P., Liu L.J., Zhang Q., Ding Z.F. (2025). Exploring Surface State and Exciplex Dominated Aggregation Induced Electrochemiluminescence of Graphene Quantum Dots Prepared via Electrochemical Exfoliation. Chemphyschem.

[71] Ye R., Xiang C., Lin J., Peng Z., Huang K., Yan Z., Cook N.P., Samuel E.L., Hwang C.-C., Ruan G. (2013). Coal as an abundant source of graphene quantum dots. Nat. Commun..

[72] Zarghami A., Dolatyari M., Mirtagioglu H., Rostami A. (2023). High-efficiency upconversion process in cobalt and neodymium doped graphene QDs for biomedical applications. Sci. Rep..

[73] Routh P., Das S., Shit A., Bairi P., Das P., Nandi A.K. (2013). Graphene quantum dots from a facile sono-fenton reaction and its hybrid with a polythiophene graft copolymer toward photovoltaic application. Appl. Mater. Interfaces.

[74] Yoo J.M., Kang J.H., Hong B.H. (2015). Graphene-based nanomaterials for versatile imaging studies. Chem. Soc. Rev..

[75] Li S., Zhou S., Li Y., Li X., Zhu J., Fan L., Yang S. (2017). Exceptionally High Payload of the IR780 Iodide on Folic Acid-Functionalized Graphene Quantum Dots for Targeted Photothermal Therapy. ACS Appl. Mater. Interfaces.

[76] Peng J., Gao W., Gupta B.K., Liu Z., Romero-Aburto R., Ge L., Song L., Alemany L.B., Zhan X., Gao G. (2012). Graphene quantum dots derived from carbon fibers. Nano Lett..

[77] Wu J., Wang P., Wang F., Fang Y. (2018). Investigation of the Microstructures of Graphene Quantum Dots (GQDs) by Surface-Enhanced Raman Spectroscopy. Nanomaterials.

[78] Zhuo S., Shao M., Lee S.-T. (2012). Upconversion and Downconversion Fluorescent Graphene Quantum Dots: Ultrasonic Preparation and Photocatalysis. ACS Nano.

[79] Peng D., Zhang L., Liang R.-P., Qiu J.-D. (2018). Rapid Detection of Mercury Ions Based on Nitrogen-Doped Graphene Quantum Dots Accelerating Formation of Manganese Porphyrin. ACS Sens..

[80] Tang L., Ji R., Cao X., Lin J., Jiang H., Li X., Teng K.S., Luk C.M., Zeng S., Hao J. (2012). Deep ultraviolet photoluminescence of water-soluble self-passivated graphene quantum dots. ACS Nano.

[81] Kwon W., Rhee S.-W. (2012). Facile synthesis of graphitic carbon quantum dots with size tunability and uniformity using reverse micelles. Chem. Commun..

[82] Ammar M.R., Galy N., Rouzaud J., Toulhoat N., Vaudey C., Simon P., Moncoffre N.J.C. (2015). Characterizing various types of defects in nuclear graphite using Raman scattering: Heat treatment, ion irradiation and polishing. Carbon.

[83] Chen S., Quan Y., Yu Y.-L., Wang J.-H. (2017). Graphene Quantum Dot/Silver Nanoparticle Hybrids with Oxidase Activities for Antibacterial Application. ACS Biomater. Sci. Eng..

[84] Low I.M., Albetran H.M., DeGiorgio M. (2020). Structural Characterization of Commercial Graphite and Graphene Materials. J. Nanotechnol. Nanomater..

[85] Zhu C., Han T.Y.-J., Duoss E.B., Golobic A.M., Kuntz J.D., Spadaccini C.M., Worsley M.A. (2015). Highly compressible 3D periodic graphene aerogel microlattices. Nat. Commun..

[86] Chang B.Y.S., Huang N.M., An’amt M.N., Marlinda A.R., Norazriena Y., Muhamad M.R., Harrison I., Lim H.N., Chia C.H. (2012). Facile hydrothermal preparation of titanium dioxide decorated reduced graphene oxide nanocomposite. Int. J. Nanomed..

[87] Rady H.S., Misbah M.H., El-Kemary M. (2023). Gram-scale synthesis of highly doped chlorine graphene quantum dots: Synthesis and photoluminescence properties. Carbon.

[88] Xiong S., He J.Q., Wang C.L. (2024). Preparation and Tribological Behavior of N-doped Graphene Oxide Quantum Dots with MoS_2_ and Al_2_O_3_ Nanocomposites as Lubricant Additive in Aqueous Glycerol. Tribol. Lett..

[89] Li H., He X., Kang Z., Huang H., Liu Y., Liu J., Lian S., Tsang C.H., Yang X., Lee S.T. (2010). Water-soluble fluorescent carbon quantum dots and photocatalyst design. Angew. Chem. Int. Ed. Engl..

[90] Pan D., Zhang J., Li Z., Wu C., Yan X., Wu M. (2010). Observation of pH-, solvent-, spin-, and excitation-dependent blue photoluminescence from carbon nanoparticles. Chem. Commun..

[91] Lu J., Yang J.-x., Wang J., Lim A., Wang S., Loh K.P. (2009). One-Pot Synthesis of Fluorescent Carbon Nanoribbons, Nanoparticles, and Graphene by the Exfoliation of Graphite in Ionic Liquids. ACS Nano.

[92] Li H., Kang Z., Liu Y., Lee S.-T. (2012). Carbon nanodots: Synthesis, properties and applications. J. Mater. Chem..

[93] Petrushenko I.K., Petrushenko K.B. (2019). Optical properties of bilayer quantum dot models based on coronene and its BN analogues with a BODIPY dye: Theoretical TD-CAM-B3LYP-D3 investigation. Spectrochim. Acta A Mol. Biomol. Spectrosc..

[94] Lin L., Rong M., Luo F., Chen D., Wang Y., Chen X. (2014). Luminescent graphene quantum dots as new fluorescent materials for environmental and biological applications. TrAC Trends Anal. Chem..

[95] Wang K., Dong J., Sun L., Chen H., Wang Y., Wang C., Dong L. (2016). Effects of elemental doping on the photoluminescence properties of graphene quantum dots. RSC Adv..

[96] Nair R.V., Thomas R.T., Mohamed A.P., Pillai S. (2020). Fluorescent turn-off sensor based on sulphur-doped graphene quantum dots in colloidal and film forms for the ultrasensitive detection of carbamate pesticides. Microchem. J..

[97] Lin L., Song X., Chen Y., Rong M., Zhao T., Wang Y., Jiang Y., Chen X. (2015). Intrinsic peroxidase-like catalytic activity of nitrogen-doped graphene quantum dots and their application in the colorimetric detection of H_2_O_2_ and glucose. Anal. Chim. Acta.

[98] Zhang L., Zhang Z.-Y., Liang R.-P., Li Y.-H., Qiu J.-D. (2014). Boron-Doped Graphene Quantum Dots for Selective Glucose Sensing Based on the “Abnormal” Aggregation-Induced Photoluminescence Enhancement. Anal. Chem..

[99] Li X., Lau S.P., Tang L., Ji R., Yang P. (2013). Multicolour light emission from chlorine-doped graphene quantum dots. J. Mater. Chem. C.

[100] Wang W., Xu S., Li N., Huang Z., Su B., Chen X. (2019). Sulfur and phosphorus co-doped graphene quantum dots for fluorescent monitoring of nitrite in pickles. Spectrochim. Acta A Mol. Biomol. Spectrosc..

[101] Yu Z., Ma W., Wu T., Wen J., Zhang Y., Wang L., He Y., Chu H., Hu M. (2020). Coumarin-Modified Graphene Quantum Dots as a Sensing Platform for Multicomponent Detection and Its Applications in Fruits and Living Cells. ACS Omega.

[102] Lee J.W., Kwak J.H., Kim J., Jang Y.K., Han J.T., Kim T.J., Hong K.S., Jeong H.J., Yang I.H.S. (2024). Highly emissive blue graphene quantum dots with excitation-independent emission via ultrafast liquid-phase photoreduction. RSC Adv..

[103] Berezin M.Y., Achilefu S. (2010). Fluorescence Lifetime Measurements and Biological Imaging. Chem. Rev..

[104] Röding M., Bradley S.J., Nydén M., Nann T. (2014). Fluorescence Lifetime Analysis of Graphene Quantum Dots. J. Phys. Chem. C.

[105] Liu F., Jang M.-H., Ha H.D., Kim J.-H., Cho Y.-H., Seo T.S. (2013). Facile Synthetic Method for Pristine Graphene Quantum Dots and Graphene Oxide Quantum Dots: Origin of Blue and Green Luminescence. Adv. Mater..

[106] Xu Q., Zhou Q., Hua Z., Xue Q., Zhang C., Wang X., Pan D., Xiao M. (2013). Single-Particle Spectroscopic Measurements of Fluorescent Graphene Quantum Dots. ACS Nano.

[107] Li D., Ushakova E.V., Rogach A.L., Qu S. (2021). Optical Properties of Carbon Dots in the Deep-Red to Near-Infrared Region Are Attractive for Biomedical Applications. Small.

[108] Das S.K., Luk C.M., Martin W.E., Tang L., Kim D.Y., Lau S.P., Richards C.I. (2015). Size and Dopant Dependent Single Particle Fluorescence Properties of Graphene Quantum Dots. J. Phys. Chem. C.

[109] Sk M.A., Ananthanarayanan A., Huang L., Lim K.H., Chen P. (2014). Revealing the tunable photoluminescence properties of graphene quantum dots. J. Mater. Chem. C.

[110] Zheng M., Jagota A., Semke E.D., Diner B.A., McLean R.S., Lustig S.R., Richardson R.E., Tassi N.G. (2003). DNA-assisted dispersion and separation of carbon nanotubes. Nat. Mater..

[111] Zhang J., Landry M.P., Barone P.W., Kim J.-H., Lin S., Ulissi Z.W., Lin D., Mu B., Boghossian A.A., Hilmer A.J. (2013). Molecular recognition using corona phase complexes made of synthetic polymers adsorbed on carbon nanotubes. Nat. Nanotech..

[112] Jeong S., Pinals R.L., Dharmadhikari B., Song H., Kalluri A., Debnath D., Wu Q., Ham M.-H., Patra P., Landry M.P. (2020). Graphene Quantum Dot Oxidation Governs Noncovalent Biopolymer Adsorption. Sci. Rep..

[113] Liu Y., Zhou S., Fan L., Fan H. (2016). Synthesis of red fluorescent graphene quantum dot-europium complex composites as a viable bioimaging platform. Microchim. Acta.

[114] Sapkota B., Benabbas A., Lin H.-Y.G., Liang W., Champion P., Wanunu M. (2017). Peptide-Decorated Tunable-Fluorescence Graphene Quantum Dots. ACS Appl. Mater. Interfaces.

[115] Wu X., Ma L., Sun S., Jiang K., Zhang L., Wang Y., Zeng H., Lin H. (2018). A versatile platform for the highly efficient preparation of graphene quantum dots: Photoluminescence emission and hydrophilicity-hydrophobicity regulation and organelle imaging. Nanoscale.

[116] Halder A., Godoy-Gallardo M., Ashley J., Feng X., Zhou T., Hosta-Rigau L., Sun Y. (2018). One-Pot Green Synthesis of Biocompatible Graphene Quantum Dots and Their Cell Uptake Studies. ACS Appl. Bio Mater..

[117] Liu R., Wu D., Feng X., Müllen K. (2011). Bottom-up Fabrication of Photoluminescent Graphene Quantum Dots with Uniform Morphology. J. Am. Chem. Soc..

[118] Nurunnabi M., Khatun Z., Nafiujjaman M., Lee D.G., Lee Y.K. (2013). Surface coating of graphene quantum dots using mussel-inspired polydopamine for biomedical optical imaging. ACS Appl. Mater. Interfaces.

[119] Iannazzo D., Ziccarelli I., Pistone A. (2017). Graphene quantum dots: Multifunctional nanoplatforms for anticancer therapy. J. Mater. Chem. B.

[120] Biswas M.C., Islam M.T., Nandy P.K., Hossain M.M. (2021). Graphene Quantum Dots (GQDs) for Bioimaging and Drug Delivery Applications: A Review. ACS Mater. Lett..

[121] Song Z.M., Gong J., Soltani R., Fauny J.D., Menard-Moyon C., Chen P., Bianco A. (2024). Cellular Impact and Biodegradability of S- and N-Doped Graphene Quantum Dots on Human Monocytes and Macrophages. Adv. Funct. Mater..

[122] Feng L.L., Wu Y.X., Zhang D.L., Hu X.X., Zhang J., Wang P., Song Z.L., Zhang X.B., Tan W. (2017). Near Infrared Graphene Quantum Dots-Based Two-Photon Nanoprobe for Direct Bioimaging of Endogenous Ascorbic Acid in Living Cells. Anal. Chem..

[123] Ratnesh R.K., Singh M.K., Kumar V., Singh S., Chandra R., Singh M., Singh J. (2024). Mango Leaves (*Mangifera indica*)-Derived Highly Florescent Green Graphene Quantum Dot Nanoprobes for Enhanced On-Off Dual Detection of Cholesterol and Fe^2+^ Ions Based on Molecular Logic Operation. ACS Appl. Bio Mater..

[124] Chen X., Tian X., Shin I., Yoon J. (2011). Fluorescent and luminescent probes for detection of reactive oxygen and nitrogen species. Chem. Soc. Rev..

[125] Nathan C., Cunningham-Bussel A. (2013). Beyond oxidative stress: An immunologist’s guide to reactive oxygen species. Nat. Rev. Immunol..

[126] Gibellini L., Pinti M., Nasi M., De Biasi S., Roat E., Bertoncelli L., Cossarizza A. (2010). Interfering with ROS metabolism in cancer cells: The potential role of quercetin. Cancers.

[127] Zhang R., Zhao J., Han G., Liu Z., Liu C., Zhang C., Liu B., Jiang C., Liu R., Zhao T. (2016). Real-time discrimination and versatile profiling of spontaneous reactive oxygen species in living organisms with a single fluorescent probe. J. Am. Chem. Soc..

[128] Kundu K., Knight S.F., Willett N., Lee S., Taylor W.R., Murthy N. (2009). Hydrocyanines: A class of fluorescent sensors that can image reactive oxygen species in cell culture, tissue, and in vivo. Angew. Chem..

[129] Liu R., Zhang L., Chen Y., Huang Z., Huang Y., Zhao S. (2018). Design of a New Near-Infrared Ratiometric Fluorescent Nanoprobe for Real-Time Imaging of Superoxide Anions and Hydroxyl Radicals in Live Cells and in Situ Tracing of the Inflammation Process in Vivo. Anal. Chem..

[130] Wibowo A., Khan M.J., Sawatdee S., Pornputthapitak W., Tuntithavornwat S., Srifa A., Posoknistakul P., Pornsuwan S., Laosiripojana N., Jiang Y.J. (2026). Structure-property relationships in saccharide-derived carbon dots: Tuning oxygen functionalities and sp2 domains for antioxidant performance. J. Colloid Interface Sci..

[131] Guo Y., Deng L., Li J., Guo S., Wang E., Dong S. (2011). Hemin−graphene hybrid nanosheets with intrinsic peroxidase-like activity for label-free colorimetric detection of single-nucleotide polymorphism. ACS Nano.

[132] Li R.Y., Xie Q.Q., Li Z.J., Zhang R.L., Yang Y.Q., Liu X.H. (2025). Synthesis of histidine, serine and folic acid-functionalized and boron and iron-doped graphene quantum dot with excellent optical behavior and peroxidase-like activity for colorimetric and fluorescence detection of H_2_O_2_ in food. Spectrochim. Acta Part A-Mol. Biomol. Spectrosc..

[133] Hua M., Wang C., Qian J., Wang K., Yang Z., Liu Q., Mao H., Wang K. (2015). Preparation of graphene quantum dots based core-satellite hybrid spheres and their use as the ratiometric fluorescence probe for visual determination of mercury(II) ions. Anal. Chim. Acta.

[134] Shao N., Zhang Y., Cheung S., Yang R., Chan W., Mo T., Li K., Liu F. (2005). Copper ion-selective fluorescent sensor based on the inner filter effect using a spiropyran derivative. Anal. Chem..

[135] Zhang L., Peng D., Liang R.P., Qiu J.D. (2015). Graphene quantum dots assembled with metalloporphyrins for “turn on” sensing of hydrogen peroxide and glucose. Chem. Eur. J..

[136] Pocernich C.B., Butterfield D.A. (2012). Elevation of glutathione as a therapeutic strategy in Alzheimer disease. Biochim. Biophys. Acta Mol. Basis Dis..

[137] Cai Q.-Y., Li J., Ge J., Zhang L., Hu Y.-L., Li Z.-H., Qu L.-B. (2015). A rapid fluorescence “switch-on” assay for glutathione detection by using carbon dots–MnO_2_ nanocomposites. Biosens. Bioelectron..

[138] Townsend D.M., Tew K.D., Tapiero H. (2003). The importance of glutathione in human disease. Biomed. Pharmacother..

[139] Yan X., Song Y., Zhu C., Song J., Du D., Su X., Lin Y. (2016). Graphene quantum dot–MnO_2_ nanosheet based optical sensing platform: A sensitive fluorescence “turn off–on” nanosensor for glutathione detection and intracellular imaging. ACS Appl. Mater. Interfaces.

[140] Zhang J., Campbell R.E., Ting A.Y., Tsien R.Y. (2002). Creating new fluorescent probes for cell biology. Nat. Rev. Mol. Cell Biol..

[141] Antoine C., Pijeira M.S.O., Ricci E., Alencar L.M.R., Santos-Oliveira R. (2022). Graphene quantum dots as bimodal imaging agent for X-ray and Computed Tomography. Eur. J. Pharm. Biopharm..

[142] Pechnikova N.A., Domvri K., Porpodis K., Istomina M.S., Iaremenko A.V., Yaremenko A.V. (2025). Carbon Quantum Dots in Biomedical Applications: Advances, Challenges, and Future Prospects. Aggregate.

[143] Yan H., Wang Q., Wang J.Y., Shang W.T., Xiong Z.Y., Zhao L.Y., Sun X.D., Tian J., Kang F.Y., Yun S.H. (2023). Planted Graphene Quantum Dots for Targeted, Enhanced Tumor Imaging and Long-Term Visualization of Local Pharmacokinetics. Adv. Mater..

[144] Das C., Sepay N., Kim T.W., Chae S., Ghosh N., Dumpala M., Choi D., Jeon S., Im J., Biswas G. (2025). Recycling Motorcycle Exhaust Soot into Fluorescent Graphene Oxide Quantum Dots for Sensing Ferrocyanide Ions and Bioimaging Cells: A Method for Waste Utilization. ACS Omega.

[145] Gao T., Wang X., Zhao J., Jiang P., Jiang F.-L., Liu Y. (2020). Bridge between Temperature and Light: Bottom-Up Synthetic Route to Structure-Defined Graphene Quantum Dots as a Temperature Probe In Vitro and in Cells. ACS Appl. Mater. Interfaces.

[146] Horgan C.C., Bergholt M.S., Nagelkerke A., Thin M.Z., Pence I.J., Kauscher U., Kalber T.L., Stuckey D.J., Stevens M.M. (2021). Integrated photodynamic Raman theranostic system for cancer diagnosis, treatment, and post-treatment molecular monitoring. Theranostics.

[147] Lin J., Chen X.Y., Huang P. (2016). Graphene-based nanomaterials for bioimaging. Adv. Drug Deliv. Rev..

[148] Ferrer-Ugalde A., Sandoval S., Pulagam K.R., Muñoz-Juan A., Laromaine A., Llop J., Tobias G., Núñez R. (2021). Radiolabeled Cobaltabis(dicarbollide) Anion-Graphene Oxide Nanocomposites for In Vivo Bioimaging and Boron Delivery. ACS Appl. Nano Mater..

[149] Handayani M., Hendrik, Abbas A., Anshori I., Mulyawan R., Satriawan A., Shalannanda W., Setianingsih C., Pingak C.T.R., Zahro Q. (2023). Development of graphene and graphene quantum dots toward biomedical engineering applications: A review. Nanotechnol. Rev..

[150] Kuo W.S., Chang C.Y., Chuang H.Y., Su P.L., Wang J.Y., Wu P.C., Kao H.F., Tseng S.W., Lin S.H., Lin Y.S. (2023). Single-sized N-functionality graphene quantum dot in tunable dual-modality near infrared-I/II illumination detection and photodynamic therapy under multiphoton nonlinear excitation. Biosens. Bioelectron..

[151] Kuo W.S., Lin Y.S., Chang C.Y., Wang J.Y., Chen P.C., Tseng S.W., Lin C.Y., Chang C.C., Wu S.R. (2026). Graphene hybrid nanoprobes for targeted microbial sensing and ultralow-energy, deep-tissue, noninvasive multiphoton imaging in the NIR-I/II region. Biosens. Bioelectron..

[152] Liang L.J., Shen X., Zhou M.D., Chen Y.J., Lu X.D., Zhang L., Wang W., Shen J.W. (2022). Theoretical Evaluation of Potential Cytotoxicity of Graphene Quantum Dot to Adsorbed DNA. Materials.

[153] Yan X., Li B., Li L.S. (2013). Colloidal graphene quantum dots with well-defined structures. Acc. Chem. Res..

[154] Ding H., Zhang F., Zhao C., Lv Y., Ma G., Wei W., Tian Z. (2017). Beyond a Carrier: Graphene Quantum Dots as a Probe for Programmatically Monitoring Anti-Cancer Drug Delivery, Release, and Response. ACS Appl. Mater. Interfaces.

[155] Yao X., Tian Z., Liu J., Zhu Y., Hanagata N. (2017). Mesoporous Silica Nanoparticles Capped with Graphene Quantum Dots for Potential Chemo–Photothermal Synergistic Cancer Therapy. Langmuir.

[156] Gómez I.J., Ovejero-Paredes K., Méndez-Arriaga J.M., Pizúrová N., Filice M., Zajicková L., Prashar S., Gómez-Ruiz S. (2023). Organotin(IV)-Decorated Graphene Quantum Dots as Dual Platform for Molecular Imaging and Treatment of Triple Negative Breast Cancer. Chem.-A Eur. J..

[157] Li Z., Fan J., Tong C., Zhou H., Wang W., Li B., Liu B., Wang W. (2019). A smart drug-delivery nanosystem based on carboxylated graphene quantum dots for tumor-targeted chemotherapy. Nanomedicine.

[158] Wang C., Chen Y., Xu Z., Chen B., Zhang Y., Yi X., Li J. (2020). Fabrication and characterization of novel cRGD modified graphene quantum dots for chemo-photothermal combination therapy. Sens. Actuators. A Phys..

[159] Xuan Y., Zhang R.-Y., Zhao D.-H., Zhang X.-S., An J., Cheng K., Hou X.-L., Song X.-L., Zhao Y.-D., Yang X.-Q. (2019). Ultrafast synthesis of gold nanosphere cluster coated by graphene quantum dot for active targeting PA/CT imaging and near-infrared laser/pH-triggered chemo-photothermal synergistic tumor therapy. Chem. Eng. J..

[160] Chen L., Hong W., Duan S., Li Y., Wang J., Zhu J. (2022). Graphene quantum dots mediated magnetic chitosan drug delivery nanosystems for targeting synergistic photothermal-chemotherapy of hepatocellular carcinoma. Cancer Biol. Ther..

[161] Monro S., Colón K.L., Yin H., Roque J., Konda P., Gujar S., Thummel R.P., Lilge L., Cameron C.G., McFarland S.A. (2018). Transition metal complexes and photodynamic therapy from a tumor-centered approach: Challenges, opportunities, and highlights from the development of TLD1433. Chem. Rev..

[162] Milenkovic M., Filimonovic M.B., Ciasca G., Milivojevic D., Filipovic J.P., Mojsin M., Jovanovic S., Markovic B.T. (2026). Combining two biocompatible singlet oxygen generators into a potent photoactive agent: Graphene quantum dots-curcuma hybrid. Mater. Res. Bull..

[163] Chen J., Keltner L., Christophersen J., Zheng F., Krouse M., Singhal A., Wang S. (2002). New technology for deep light distribution in tissue for phototherapy. J. Cancer.

[164] Resch-Genger U., Grabolle M., Cavaliere-Jaricot S., Nitschke R., Nann T. (2008). Quantum dots versus organic dyes as fluorescent labels. Nat. Methods.

[165] Torchi A., Simonelli F., Ferrando R., Rossi G. (2017). Local Enhancement of Lipid Membrane Permeability Induced by Irradiated Gold Nanoparticles. ACS Nano.

[166] Zhang S., Guo W., Wei J., Li C., Liang X.-J., Yin M. (2017). Terrylenediimide-based intrinsic theranostic nanomedicines with high photothermal conversion efficiency for photoacoustic imaging-guided cancer therapy. ACS Nano.

[167] Ge J., Jia Q., Liu W., Guo L., Liu Q., Lan M., Zhang H., Meng X., Wang P. (2015). Red-emissive carbon dots for fluorescent, photoacoustic, and thermal theranostics in living mice. Adv. Mater..

[168] Vankayala R., Lin C.-C., Kalluru P., Chiang C.-S., Hwang K.C. (2014). Gold nanoshells-mediated bimodal photodynamic and photothermal cancer treatment using ultra-low doses of near infra-red light. Biomaterials.

[169] Hwang S., Nam J., Jung S., Song J., Doh H., Kim S. (2014). Gold nanoparticle-mediated photothermal therapy: Current status and future perspective. Nanomedicine.

[170] Liu Y., Bhattarai P., Dai Z., Chen X. (2019). Photothermal therapy and photoacoustic imaging via nanotheranostics in fighting cancer. Chem. Soc. Rev..

[171] Pansare V.J., Hejazi S., Faenza W.J., Prud’homme R.K. (2012). Review of long-wavelength optical and NIR imaging materials: Contrast agents, fluorophores, and multifunctional nano carriers. Chem. Mater..

[172] Zhang C., Ji X., Zhang Y., Zhou G., Ke X., Wang H., Tinnefeld P., He Z. (2013). One-pot synthesized aptamer-functionalized CdTe: Zn2+ quantum dots for tumor-targeted fluorescence imaging in vitro and in vivo. Anal. Chem..

[173] Robinson J.T., Tabakman S.M., Liang Y., Wang H., Sanchez Casalongue H., Vinh D., Dai H. (2011). Ultrasmall reduced graphene oxide with high near-infrared absorbance for photothermal therapy. J. Am. Chem. Soc..

[174] Lu W., Singh A.K., Khan S.A., Senapati D., Yu H., Ray P.C. (2010). Gold nano-popcorn-based targeted diagnosis, nanotherapy treatment, and in situ monitoring of photothermal therapy response of prostate cancer cells using surface-enhanced Raman spectroscopy. J. Am. Chem. Soc..

[175] Wang C., Cheng L., Liu Z. (2011). Drug delivery with upconversion nanoparticles for multi-functional targeted cancer cell imaging and therapy. Biomaterials.

[176] Bates P.J., Kahlon J.B., Thomas S.D., Trent J.O., Miller D.M. (1999). Antiproliferative activity of G-rich oligonucleotides correlates with protein binding. J. Biol. Chem..

[177] Reyes-Reyes E.M., Teng Y., Bates P.J. (2010). A new paradigm for aptamer therapeutic AS1411 action: Uptake by macropinocytosis and its stimulation by a nucleolin-dependent mechanism. J. Cancer Res..

[178] Wang X., Sun X., He H., Yang H., Lao J., Song Y., Xia Y., Xu H., Zhang X., Huang F. (2015). A two-component active targeting theranostic agent based on graphene quantum dots. J. Mater. Chem. B.

[179] Acharya A., Das I., Chandhok D., Saha T. (2010). Redox regulation in cancer: A double-edged sword with therapeutic potential. Oxid. Med. Cell Longev..

[180] Chaiswing L., St. Clair W.H., St. Clair D.K. (2018). Redox Paradox: A novel approach to therapeutics-resistant cancer. Antioxid. Redox Signal..

[181] Yang Y., Chen S., Liu L., Li S., Zeng Q., Zhao X., Li H., Zhang Z., Bouchard L.-S., Liu M. (2017). Increasing Cancer Therapy Efficiency through Targeting and Localized Light Activation. ACS Appl. Mater. Interfaces.

[182] Kurniawan D., Mathew J., Rahardja M.R., Pham H.P., Wong P.C., Rao N.V., Ostrikov K., Chiang W.H. (2023). Plasma-Enabled Graphene Quantum Dot Hydrogels as Smart Anticancer Drug Nanocarriers. Small.

[183] Das N., Srivastava R., Roy S., De A.K., Kar R.K. (2025). Physico-chemical properties and biological evaluation of graphene quantum dots for anticancer drug susceptibility. Colloids Surf. B-Biointerfaces.

[184] Poursadegh H., Sayar M.A., Nickabadi S., Golmohammadi B., Rostami H. (2026). Synthesis of polymer composite films reinforced with MOFs/magnetic graphene quantum dots for microwave absorption. Colloids Surf. A-Physicochem. Eng. Asp..

[185] Milenkovic M., Jovanovic S., Markovic Z., Ciasca G., Di Santo R., Mead J.L., Mojsin M., Dojcinovic B., Milivojevic D., Markovic B.T. (2025). Graphene quantum dots enhanced with gold nanoparticles for advanced antibacterial applications. J. Drug Deliv. Sci. Technol..

[186] Rahardja M.R., Kurniawan D., Laysandra T., Chiang W.H. (2025). Graphene Quantum Dot-Cross-Linked Bioresource Hydrogels for Selective Molecular Capture in Therapeutic Loading and Environmental Remediation. ACS Appl. Nano Mater..

[187] Raj S., Vijaya J.J., Fermi J.J. (2025). A Sustainable Green Synthesis of Graphene Quantum Dots Using Groundnut and Medicago sativa Oil Cakes for Biomedical Applications. Bionanoscience.

[188] Kadyan P., Saini J., Singh P., Arasu P.T., Kataria S.K. (2025). Unveiling antidiabetic and antioxidant activities of biogenically synthesized red fluorescent graphene quantum dots using Bauhinia variegata L. leaves. Discov. Sustain..

[189] Singh S., Gupta A.K. (2011). Nitric oxide: Role in tumour biology and iNOS/NO-based anticancer therapies. Cancer Chemother. Pharmacol..

[190] Ignarro L.J. (2000). Nitric Oxide: Biology and Pathobiology.

[191] Li Y.-H., Guo M., Shi S.-W., Zhang Q.-L., Yang S.-P., Liu J.-G. (2017). A ruthenium-nitrosyl-functionalized nanoplatform for the targeting of liver cancer cells and NIR-light-controlled delivery of nitric oxide combined with photothermal therapy. J. Mater. Chem. B.

[192] Chen L., Yang S., Cui X., Liu Z., Wang H., Li Y., He P., Wang M., Ding G. (2026). Machine learning-driven advances in carbon-based quantum dots: Opportunities accompanied by challenges. Responsive Mater..

[193] Das R., Paria A., Giri P.K. (2025). Machine learning framework for selective and sensitive metal ion sensing with nitrogen-doped graphene quantum dots heterostructure. Carbon.

